# Quercetin-loaded mesoporous nano-delivery system remodels osteoimmune microenvironment to regenerate alveolar bone in periodontitis via the miR-21a-5p/PDCD4/NF-κB pathway

**DOI:** 10.1186/s12951-024-02352-4

**Published:** 2024-03-06

**Authors:** Shi-Yuan Yang, Yue Hu, Ran Zhao, Yu-Ning Zhou, Yu Zhuang, Yan Zhu, Xiao-Li Ge, Ting-Wei Lu, Kai-Li Lin, Yuan-Jin Xu

**Affiliations:** 1 Department of Oral Surgery, Shanghai Ninth People’s Hospital, Shanghai Jiao Tong University School of Medicine, 639 Zhizaoju Road, Shanghai, 200011 China; 2 College of Stomatology, National Center for Stomatology, National Clinical Research Center for Oral Diseases, Shanghai Key Laboratory of Stomatology, Shanghai Jiao Tong University, Shanghai Research Institute of Stomatology, Shanghai, China; 3 Department of Oral Mucosal Diseases, Shanghai Ninth People’s Hospital, Shanghai Jiao Tong University School of Medicine, Shanghai, China; 4 Department of Oral and Cranio-Maxillofacial Surgery, Shanghai Ninth People’s Hospital, Shanghai Jiao Tong University School of Medicine, Shanghai, China

**Keywords:** Periodontitis, Quercetin, Alveolar bone regeneration, Osteoimmunomodulation, Mesoporous bioactive glass

## Abstract

**Background:**

Impaired osteo-/angiogenesis, excessive inflammation, and imbalance of the osteoimmune homeostasis are involved in the pathogenesis of the alveolar bone defect caused by periodontitis. Unfortunately, there is still a lack of ideal therapeutic strategies for periodontitis that can regenerate the alveolar bone while remodeling the osteoimmune microenvironment. Quercetin, as a monomeric flavonoid, has multiple pharmacological activities, such as pro-regenerative, anti-inflammatory, and immunomodulatory effects. Despite its vast spectrum of pharmacological activities, quercetin’s clinical application is limited due to its poor water solubility and low bioavailability.

**Results:**

In this study, we fabricated a quercetin-loaded mesoporous bioactive glass (Quercetin/MBG) nano-delivery system with the function of continuously releasing quercetin, which could better promote the bone regeneration and regulate the immune microenvironment in the alveolar bone defect with periodontitis compared to pure MBG treatment. In particular, this nano-delivery system effectively decreased injection frequency of quercetin while yielding favorable therapeutic results. In view of the above excellent therapeutic effects achieved by the sustained release of quercetin, we further investigated its therapeutic mechanisms. Our findings indicated that under the periodontitis microenvironment, the intervention of quercetin could restore the osteo-/angiogenic capacity of periodontal ligament stem cells (PDLSCs), induce immune regulation of macrophages and exert an osteoimmunomodulatory effect. Furthermore, we also found that the above osteoimmunomodulatory effects of quercetin via macrophages could be partially blocked by the overexpression of a key microRNA——miR-21a-5p, which worked through inhibiting the expression of PDCD4 and activating the NF-κB signaling pathway.

**Conclusion:**

In summary, our study shows that quercetin-loaded mesoporous nano-delivery system has the potential to be a therapeutic approach for reconstructing alveolar bone defects in periodontitis. Furthermore, it also offers a new perspective for treating alveolar bone defects in periodontitis by inhibiting the expression of miR-21a-5p in macrophages and thereby creating a favorable osteoimmune microenvironment.

**Graphical Abstract:**

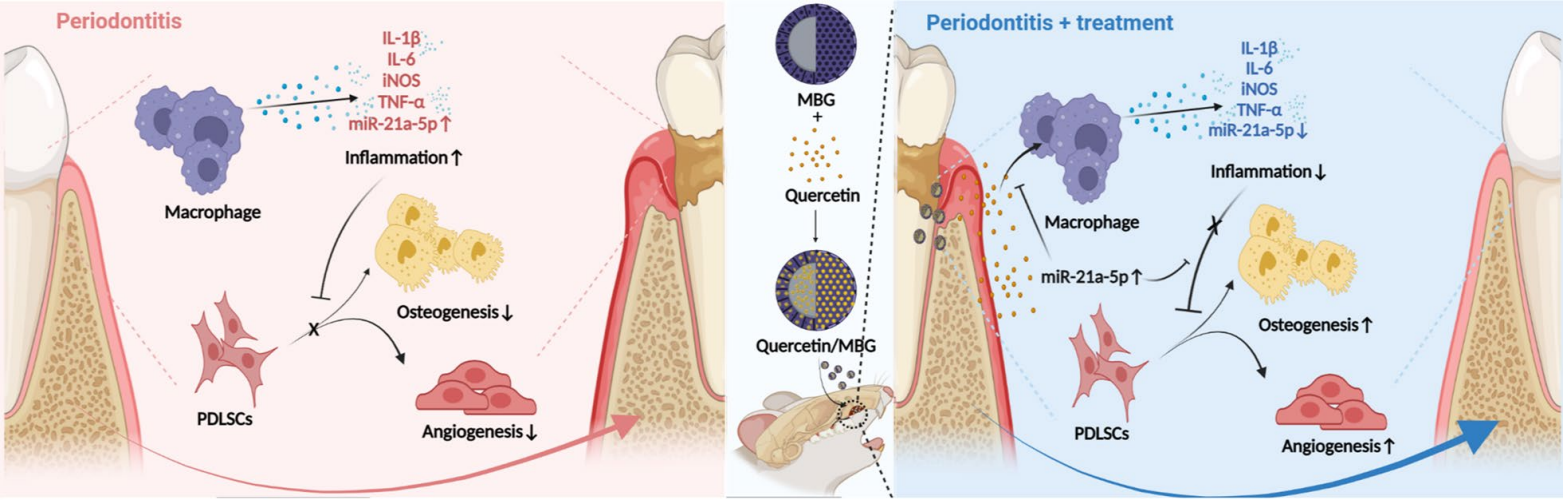

**Supplementary Information:**

The online version contains supplementary material available at 10.1186/s12951-024-02352-4.

## Background

Periodontitis is the second most common oral disease worldwide, with a prevalence exceeding 40% [1]. As a chronic, destructive inflammatory disease affecting the periodontal supporting tissues, loss of alveolar bone is a key feature of the disease, which may eventually lead to tooth loss [2]. Studies on pathological mechanisms have demonstrated that impaired bone repair capacity and the imbalanced osteoimmune microenvironment are major impediments to regenerating damaged alveolar bone in periodontitis [3]. Currently, the clinical treatment of alveolar bone defects caused by periodontitis was primarily based on local irritant removal, antibiotic therapy, and alveolar bone surgery, while these therapies could not simultaneously regenerate alveolar bone and remodel optimal osteoimmune microenvironment [1, 4]. Thus, a novel therapeutic strategy is required to stimulate alveolar bone regeneration and simultaneously manage the osteoimmune microenvironment to achieve in situ alveolar bone reconstruction in periodontitis.

PDLSCs, a type of mesenchymal stem cells (MSCs), are closely associated with alveolar bone repair, which have promising potential in the field of periodontal regeneration [5]. The osteo-/angiogenesis of PDLSCs could be hindered by the chronic inflammation in periodontitis, leading to periodontal regeneration disorder [6]. As a key immune cell in the development of periodontitis, macrophages contribute significantly to osteoimmune homeostasis [7]. Generally, macrophages are typically classified into two phenotypes based on their activation status and function: the pro-inflammatory M1 phenotype and the anti-inflammatory M2 phenotype. During the initial phase of the inflammatory response in periodontitis, the activation of M1 phenotype is beneficial for tissue defense, but if this state persists beyond this period, it would lead to irreversible destruction of periodontal tissue [8]. Excessive M1 polarization of macrophages, as observed in periodontitis-related studies, worsened local immune disorders and led to destruction of periodontal tissue, while also further weakening the already delicate osteo-/angiogenesis capacity of PDLSCs and thereby exacerbating the osteoimmune microenvironment imbalance [9–11]. Therefore, restoring osteo-/angiogenic differentiation of PDLSCs and remodeling osteoimmune homeostasis via macrophages were cytologically critical for repairing alveolar bone defects in periodontitis.

Quercetin, a natural polyphenolic compound found in various plants, has several pharmacological effects including promoting osteo-/angiogenesis, anti-inflammation and immune regulation [12–15]. Our previous researches revealed that quercetin could promote the osteo-/angiogenic differentiation of bone mesenchymal stem cells (BMSCs) under osteoporosis environment and inhibit the inflammatory response in osteoarthritis [16, 17]. Although quercetin has been found to inhibit inflammatory alveolar bone absorption in experimental rat and murine periodontitis models, there is a lack of in-depth investigation into its regenerative capacity and specific cytological mechanism [18–20]. In summary, it is uncertain if quercetin can regenerate alveolar bone in periodontitis microenvironment and further investigation is necessary.

However, as a monomeric small molecule drug, quercetin's poor water solubility and low bioavailability limit its clinical use [12]. Thus, it is essential to create an effective drug delivery system that can sustain stable and suitable concentrations of quercetin in targeted tissues for clinical use. Recently, MBG nanoparticles are being considered as potential drug carriers for bone defect sites, due to their high porosity, specific surface area, and strong ability to stimulate bone tissue regeneration [21]. Furthermore, researches indicated that when MBG was utilized as a drug delivery system for bone repair, it did not elicit an unfavorable immune response in macrophages [22, 23]. Hence, it is assumed that the Quercetin/MBG nano-delivery system may have potential application in the treatment of alveolar bone defects in periodontitis.

MicroRNAs (miRNAs) are a class of evolutionarily conserved non-coding small RNAs that regulate gene expression at the translational level [24]. In recent years, many studies have reported a close relationship between periodontitis and miRNAs, such as miR-21、miR-144、miR-146a、miR-128 and etc. [25]. Furthermore, it has been reported that quercetin can induce various miRNAs to promote osteogenic differentiation as well as modulate the immune response [26–28]. Therefore, it is hypothesized that miRNAs play a pivotal role in the osteoimmunoregulatory effects of quercetin on periodontitis.

We confirmed that the Quercetin/MBG nano-delivery system consistently released quercetin and resulted in better alveolar bone regeneration and osteoimmune microenvironment remodeling compared to pure MBG. Then, we investigated the cytological mechanism by which quercetin intervened in regenerating alveolar bone under the periodontitis microenvironment. We observed that quercetin not only restored the osteo-/angiogenic capacity of PDLSCs, but also reprogrammed macrophages through the miR-21a-5p/PDCD4/NF-κB pathway thereby remodeling the osteoimmune microenvironment. In summary, this study suggests that the quercetin-loaded mesoporous nano-delivery system holds potential as a clinical therapy for repairing alveolar bone defects in periodontitis. Moreover, manipulating miR-21a-5p in macrophages to remodel the periodontal osteoimmune microenvironment offers a novel therapeutic strategy for rebuilding the alveolar bone in periodontitis.

## Results

### Quercetin sustained-release system strengthened alveolar bone regeneration and relieved local inflammation in a rat alveolar bone defect model with periodontitis

In recent decades, it has been demonstrated that MBGs as drug carriers have the potential to repair bone defects, augment local drug concentrations, improve drug efficacy, reduce dosing frequency, and minimize side effects [29, 30]. Quercetin, as a hydrophobic biologically active agent with low oral bioavailability and broad first-pass effect. To improve local bioavailability and achieve long-term sustained release of quercetin at sites of periodontal damage with periodontitis. To improve local bioavailability and achieve long-term sustained release of quercetin at sites of periodontal damage with periodontitis, we combined MBG and quercetin for sustained release of insoluble quercetin.

**Fig. 1:**
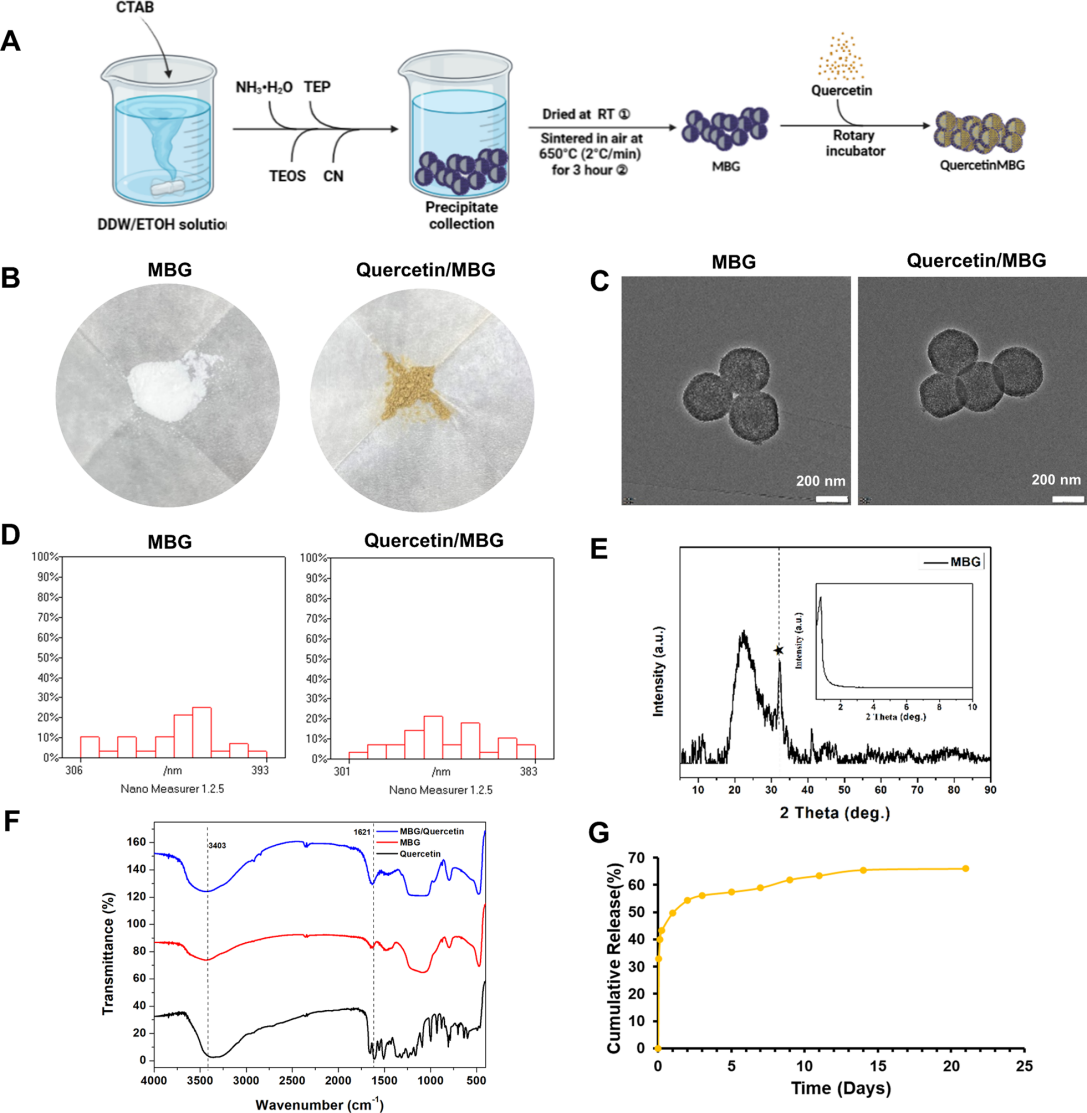
Characterization of Quercetin/MBG nano-delivery system. **A** Scheme of preparation protocol for MBG and Quercetin/MBG. **B**–**D** Visual inspection, TEM, and particle size distribution diagram of MBG and Quercetin/MBG. **E** Wide-angle and wide-angle XRD of MBG. **F** FITR of MBG and Quercetin/MBG. **G** Drug release curve of Quercetin/MBG

Figure 1A showed the preparation process of MBG and Quercetin/MBG. Figure 1B showed that the loading of quercetin caused the originally white MBG to take on a ginger color. Transmission electron microscopy (TEM) detection confirmed that MBG had a hollow microsphere morphology with mesoporous shells, and the addition of quercetin had little effect on the morphological changes of MBG (Fig. 1C). And compared to diameter of MBG (349.73 ± 43.29 nm), the diameter of Quercetin/MBG (343.27 ± 41.62 nm) changed little, indicating that quercetin was loaded in the inner core of MBG (Fig. 1D). As shown in Fig. 1E, wide-angle X-ray diffraction (XRD) spectrum indicated a composition of amorphous silica of MBG and small-angle XRD spectrum was applied to further confirm the mesoporous structure. Besides, due to the formation of the carbonate hydroxyapatite, a strong peak was seen at 2θ = 32° in the spectrum [31]. The fourier transform infrared spectrometer (FTIR) spectra in Fig. 1F showed that Quercetin/MBG contained characteristic peaks of quercetin and MBG, indicating successful loading of quercetin into MBG. In addition, the loading capacity and encapsulation efficiency of quercetin were measured to be 320.25 mg/g and 8.54%, respectively. The release profile of Quercetin/MBG nano-delivery system showed an initial burst of release within three hours, followed by sustained release over a span of 21 days (Fig. 1G).

**Fig. 2:**
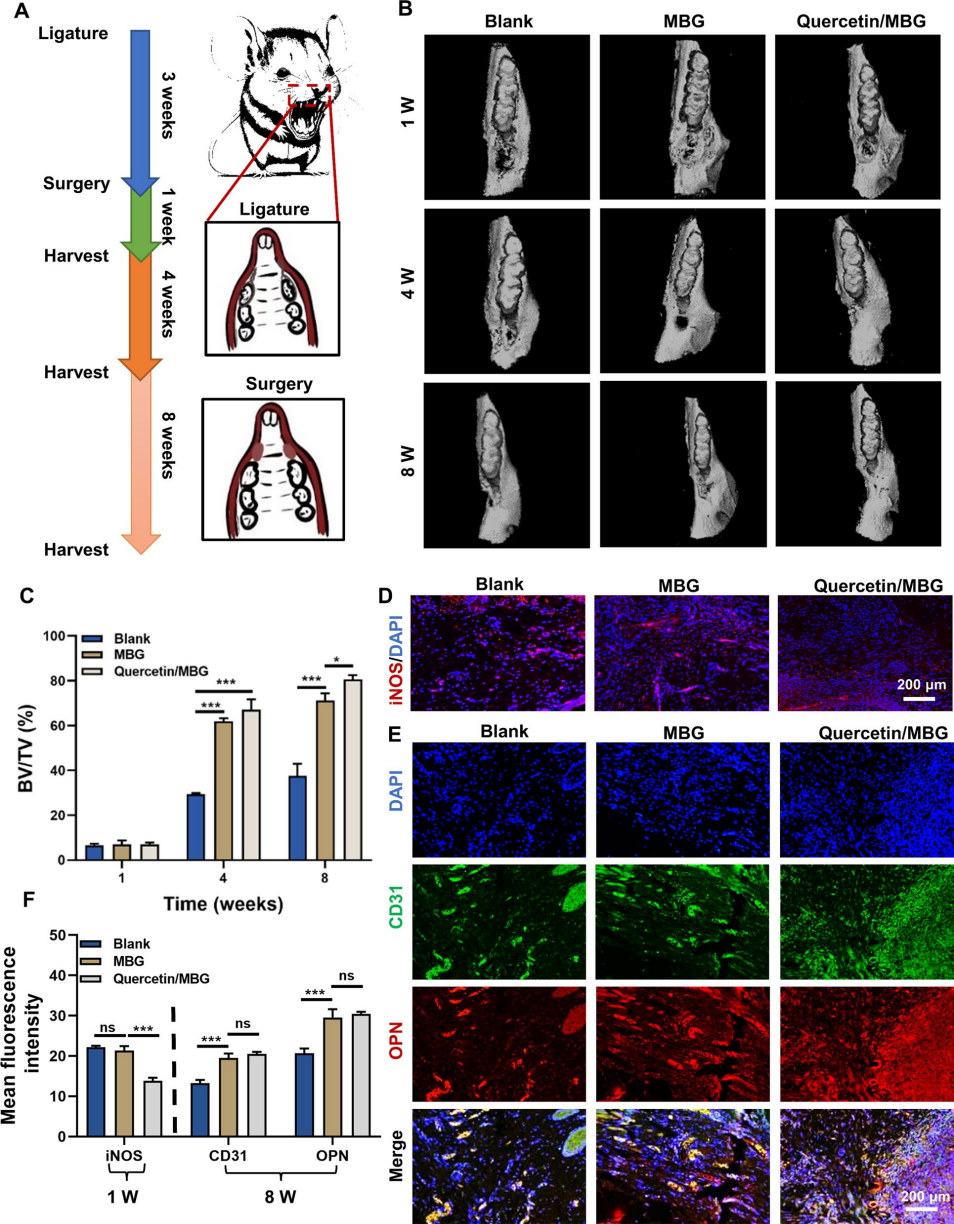
Quercetin sustained-release system strengthened alveolar bone regeneration and relieved local inflammation in a rat alveolar bone defect model with periodontitis. **A** Scheme illustration of the experimental design used for the animal study. **B** Micro-CT reconstruction images of the alveolar bone tissue. **C** Quantitative analysis of the newly formed alveolar bone tissue. **D** Immunofluorescence staining of iNOS in samples collected in 1 week. **E** Immunofluorescence staining of CD31 and OPN in samples collected in 8 weeks. **F** Quantitative analysis of the mean fluorescence intensity of iNOS, CD31and OPN according to the immunostaining patterns shown in (**D** and **E**). (ns, no significant difference; ^*^p < 0.05; ^**^p < 0.01; ^***^p < *0.001; Data are represented as the mean* ± *SEM, n* = *3)*

Figure 2A illustrated the modeling process of the rat alveolar bone defect model with periodontitis. Figure 2B presented Micro-CT reconstruction images that indicated the non-significant regeneration of bone in all groups at 1 week. However, there was a significant increase in bone mass at 4 and 8 weeks in both the MBG and Quercetin/MBG groups, with a greater increase observed in the Quercetin/MBG group. Results of bone volume/tissue volume (BV/TV) were consistent with prior Micro-CT reconstruction analyses (Fig. 2C). Histological analysis (H&E and Masson staining) of 8 week samples confirmed that quercetin supplementation improved the promotion of alveolar bone regeneration by MBG (Additional file 1: Fig. S1A, B). The immunofluorescence staining result of the 1 week samples showed high expression of inducible nitric oxide synthase (iNOS) in both the Blank and MBG groups, but no statistically significant difference was observed between these two groups. Conversely, the expression of iNOS was significantly reduced in the Quercetin/MBG group, indicating the commendable anti-inflammatory properties of quercetin. To evaluate whether continuous release of quercetin could promote osteo-/angiogenesis, we performed immunofluorescent staining for osteogenic and angiogenic markers (OPN and CD31). The study results indicated that the expression of OPN and CD31 was higher in the nanoparticle-treated group compared to the blank group, but no significant difference was observed between the MBG group and the Quercetin/MBG group (Fig. 2D–F).

In conclusion, our findings suggested that quercetin could effectively promote bone regeneration in areas of periodontal alveolar bone defects while controlling periodontal inflammation. We then investigated the underlying mechanism by which quercetin promoted alveolar bone regeneration and regulated inflammation in the periodontitis microenvironment.

### Quercetin performed pro-osteo-/angiogenesis and anti-inflammation of PDLSCs under periodontitis microenvironment

To assess the cytotoxicity of quercetin on PDLSCs, the cells were subjected to different concentrations of quercetin (0.5, 1, 2, and 4 µM) for 1, 4 and 7 days. Results from the cell counting kit-8 (CCK-8) assay indicated that quercetin had a significant cytotoxic effect at concentrations up to 4 µM on day 4 and 7, regardless of the absence or presence of the lipopolysaccharide derived from P. gingivalis (Pg.LPS) (Additional file 1: Fig. S2A, B). Additionally, the use of live/dead cell staining confirmed that quercetin (4 µM) significantly increased the number of dead cells under inflammatory environment (Additional file 1: Fig. S2C). Based on these findings, we selected quercetin concentrations of 0.5, 1, and 2 µM for further experiments.

**Fig. 3:**
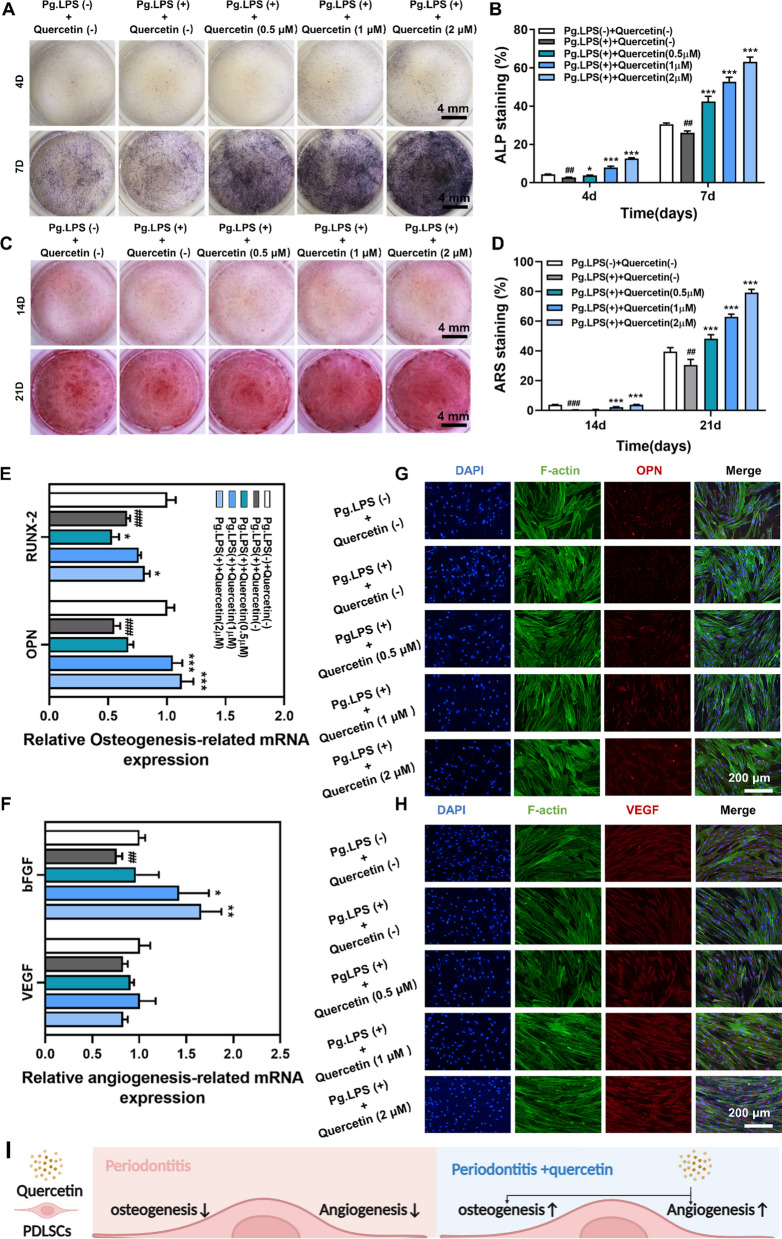
Quercetin performed pro-osteo-/angiogenesis and anti-inflammation of PDLSCs under periodontitis microenvironment. **A**, **B** ALP staining and its quantitative results after incubation of PDLSCs with quercetin for 4 and 7 days under periodontitis microenvironment. **C**, **D** ARS staining and its quantitative results after incubation of PDLSCs with quercetin for 14 and 21 days under periodontitis microenvironment. **E**–**G** Effect of quercetin on the expression of osteogenic-related genes (OPN and RUNX-2) and angiogenic-related genes (VEGF and bFGF) of PDLSCs through qRT-PCR. **H** Effect of quercetin on the expression of osteogenic-related protein (OPN) and angiogenic-related protein (VEGF) of PDLSCs through immunofluorescence staining. **I** Schematic of the pharmacological mechanism of quercetin on PDLSCs under periodontitis microenvironment. *(*^*#*^*p* < *0.05, *^*##*^*p* < *0.01 and *^*###*^*p* < *0.001 compared to Pg.LPS (-)* + Quercetin (-) group, ^*^p < 0.05, ^**^p < *0.01 and* ^***^p < *0.001 compared to Pg.LPS (* +*)* + *Quercetin (-) group; Data are represented as the mean* ± *SEM, n* = *3)*

In addition, the osteo-/angiogenic potential of PDLSCs was evaluated at varying concentrations (0.5, 1 and 2 µM) in the presence of Pg.LPS (1 μg/mL). As demonstrated in Fig. 3A–D, the Alkaline phosphatase (ALP) and Alizarin red S (ARS) staining revealed diminished osteogenic capacity in the presence of Pg.LPS, while quercetin exhibited a concentration-dependent upregulation of the osteogenic capacity, exhibiting the highest ALP activity and the greatest number of calcium nodules in the Pg.LPS ( +) + Quercetin (2 µM) group. Furthermore, the expression of osteogenic-related genes (OPN and RUNX family transcription factor-2, RUNX-2) and angiogenic-related gene (Basic fibrobast growth factor, bFGF) revealed by qRT-PCR analysis as well as the expression of osteogenic-related protein (OPN) and angiogenic-related protein (Vascular endothelial growth factor, VEGF) detected through immunofluorescence analysis showed a similar trend to that observed in the ALP and ARS experiments, while the expression of VEGF did not exhibit a significant difference among all groups at the gene level (Fig. 3E–H). Furthermore, the Western blot analysis revealed that the addition of Pg.LPS inhibited the expression of VEGF protein, whereas the addition of quercetin increased its expression in a concentration-dependent manner (Additional file 1: Fig. S3A-B). These results suggested that quercetin had the potential to promote osteo-/angiogenic abilities of PDLSCs (Fig. 3I).

Furthermore, we evaluated the anti-inflammatory potential of quercetin in PDLSCs as illustrated in Additional file 1: Fig. S4A–C, which was conducive to remodeling the periodontal microenvironment and promoting alveolar bone regeneration. The results of qRT-PCR and immunofluorescence staining respectively indicated that quercetin could impede inflammatory responses at both the gene and protein levels. Further, quercetin was found to maximally suppress the activated immune response induced by Pg.LPS at a concentration of 2 µM.

This part confirmed that quercetin significantly promoted osteo-/angiogenesis, while simultaneously suppressing inflammation in PDLSCs, indicating the exceptional osteo-/angiogenic and anti-inflammatory properties of quercetin.

### Quercetin performed anti-inflammation of macrophages under periodontitis microenvironment

**Fig. 4:**
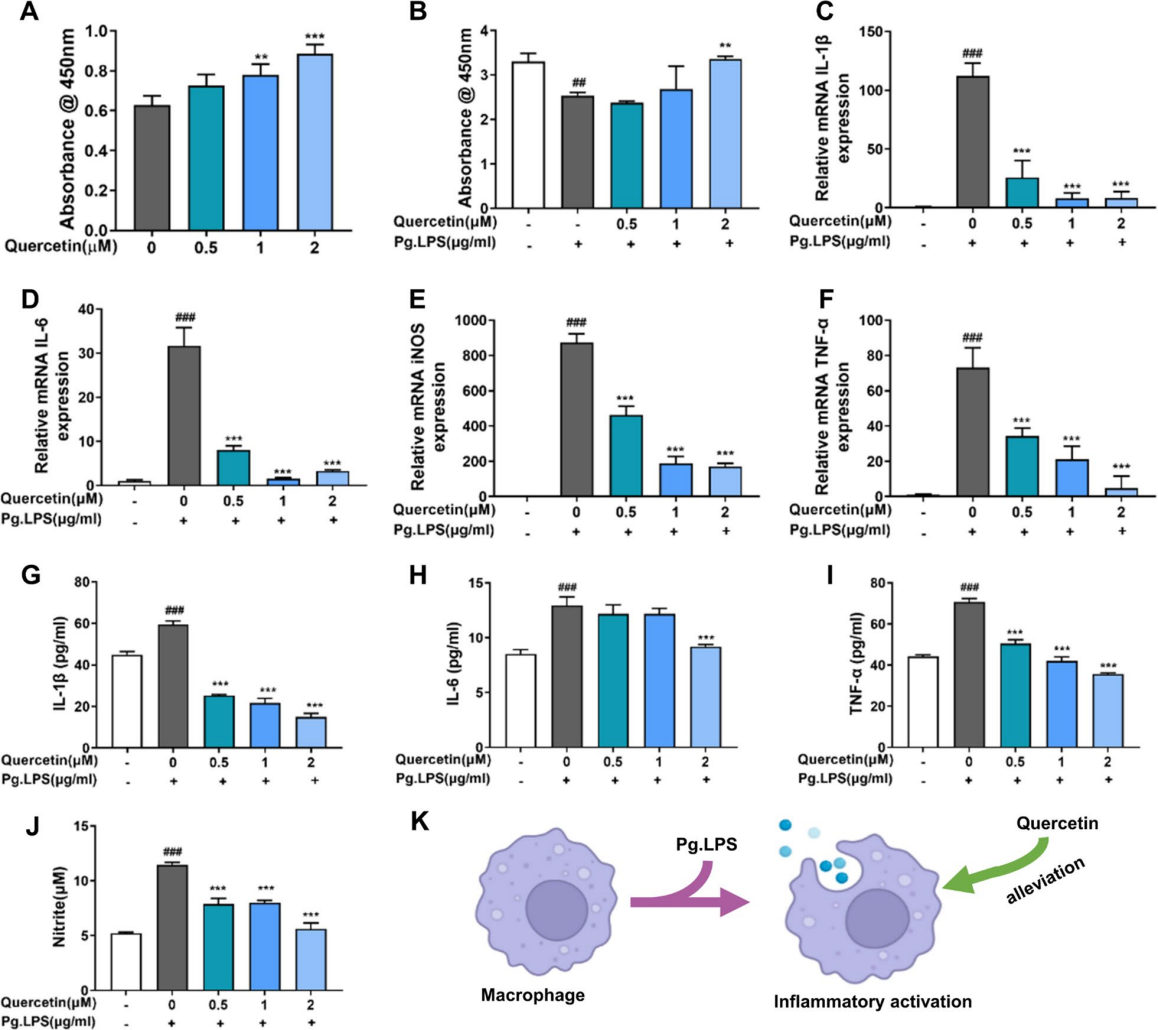
Quercetin performed anti-inflammation of macrophages under periodontitis microenvironment. **A**, **B** Proliferation results of RAW264.7 cells incubated with quercetin for 1 day under physiological and periodontitis environment through CCK-8 assay. **C**–**F** Effect of quercetin on the expression of inflammatory genes (IL-1β, IL-6, iNOS and TNF-α) of RAW264.7 through qRT-PCR. **G**–**J** Effect of quercetin on the expression of inflammatory cytokines (IL-1β, IL-6, iNOS and TNF-α) of RAW264.7 through Elisa tests. **K** Schematic of the pharmacological mechanism of quercetin on macrophages under periodontitis microenvironment. (^*#*^*p* < *0.05, *^*##*^*p* < *0.01 and *^*###*^*p* < *0.001 compared to Pg.LPS (-)* + Quercetin (-) group, ^*^p < 0.05, ^**^p < *0.01 and* ^***^p < *0.001 compared to Pg.LPS (* +*)* + *Quercetin (-) group; Data are represented as the mean* ± *SEM, n* = *3)*

During this study, we cultured RAW264.7 cells with different concentrations of quercetin (0.5–2 μM) and Pg.LPS (1 μg/mL) to examine cell viability as well as gene and protein levels of Interleukin-1β (IL-1β), Interleukin-6 (IL-6), iNOS and Tumor necrosis factor-α (TNF-α). The data presented in Fig. 4A, B indicated that various concentrations of quercetin (0.5, 1 and 2 µM) exhibited no cytotoxic effects in the absence or presence of Pg.LPS. Additionally, our observations revealed that exposure to Pg.LPS notably heightened the mRNA level and cytokine release of IL-1β, IL-6, iNOS and TNF-α in RAW 264.7 cells, which were decreased by quercetin treatment in a dose-dependent manner (Fig. 4C–J). The findings of this study displayed that quercetin could suppress Pg.LPS-induced inflammation in RAW264.7 cells and 2 µM of quercetin was selected as the focus concentration for the subsequent experiments (Fig. 4K).

### Anti-inflammatory mechanism of quercetin on macrophages under periodontitis microenvironment

#### Quercetin hindered the inflammatory reaction in Pg.LPS -stimulated macrophages through decreasing the expression of miR-21a-5p

**Fig. 5:**
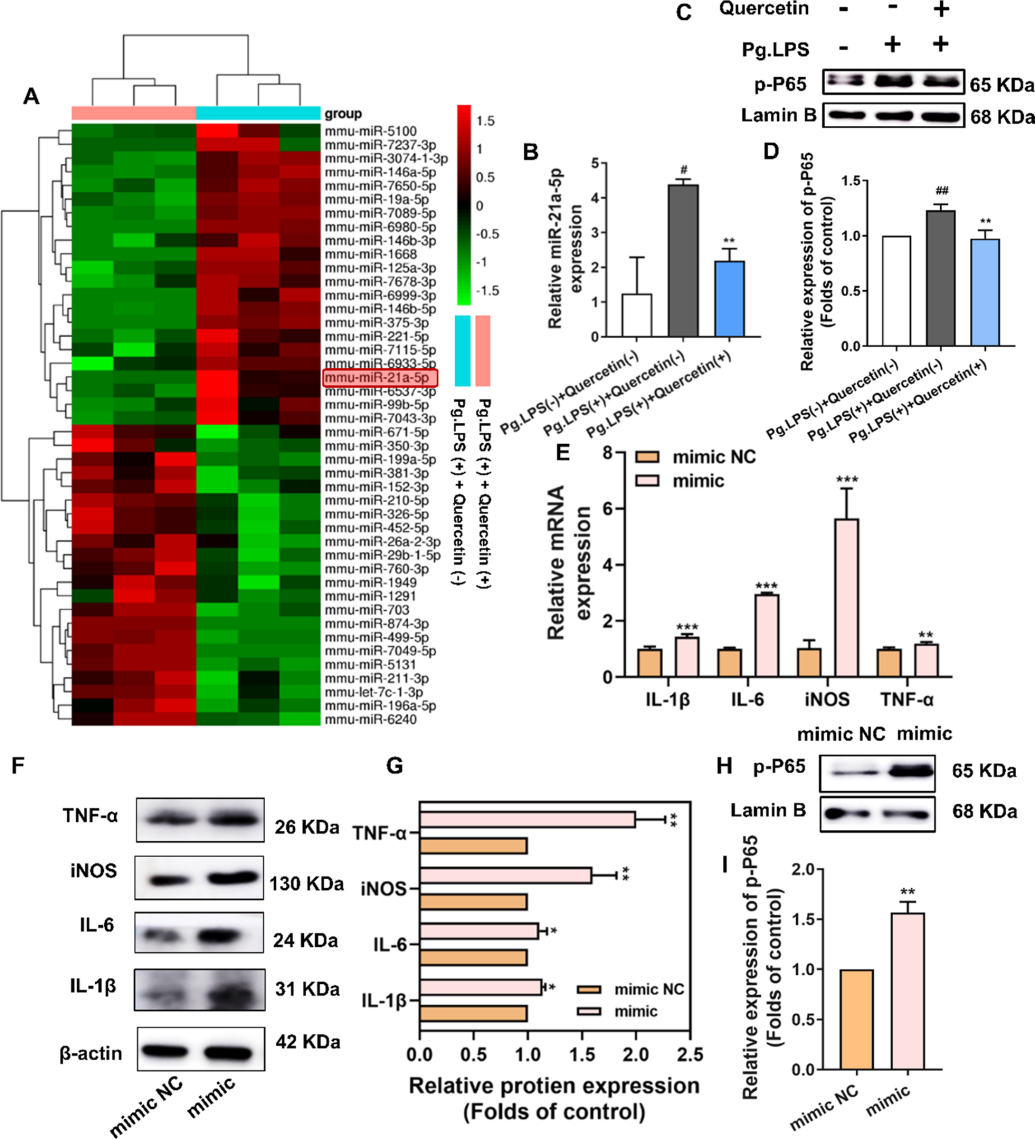
Quercetin hindered the inflammatory reaction in Pg.LPS-stimulated macrophages through decreasing the expression of miR-21a-5p. **A** Heatmaps of differential genes between Pg.LPS ( +) + Quercetin (-) and Pg.LPS ( +) + Quercetin ( +). **B** Gene expression of miR-21a-5p among Pg.LPS (-) + Quercetin (-), Pg.LPS ( +) + Quercetin (-) and Pg.LPS ( +) + Quercetin ( +) groups through qRT-PCR. **C**, **D** Western blot and its quantitative analysis of p-P65 following incubation of macrophages with quercetin for 1 day. **E** The expression of inflammatory genes (IL-1β, IL-6, iNOS and TNF-α) through qRT-PCR in macrophages transfected with miR-21a-5p mimic NC and miR-21a-5p mimic. **F**–**I** The expression of inflammatory proteins (IL-1β, IL-6, iNOS and TNF-α) and p-P65 through western blot in RAW264.7 transfected with miR-21a-5p mimic NC and miR-21a-5p mimic. *(In *Fig. 5B–D*, *^*#*^*p* < *0.05,* ^*##*^*p* < *0.01 and *^*###*^*p* < *0.001 compared to Pg.LPS (-)* + Quercetin (-) group and ^*^p < 0.05, ^**^p < *0.01 and* ^***^p < *0.001 compared to Pg.LPS (* +*)* + *Quercetin (-) group; In *Fig.  5E–I, *p < 0.05, **p < 0.01 and ***p < *0.001; Data are represented as the mean* ± *SEM, n* = *3)*

To determine the mechanism, we conducted a miRNA microarray assay to assess whether quercetin could impact miRNA expression in RAW264.7 cells. Supervised hierarchical clustering analysis identified 224 differentially expressed miRNAs (fold-change ≥ 1.5 and false discovery rate [FDR] < 5%). Out of the 224 miRNAs identified, 22 miRNAs were observed to be downregulated in RAW264.7 cells in the presence of quercetin (Fig. 5A). Given the correlation between miR-21a-5p and inflammation, we selected this miRNA for additional research. Further validation was conducted using qRT-PCR test to confirm that quercetin could inhibit the elevation of miR-21a-5p expression induced by Pg.LPS (Fig. 5B). Additionally, performing Gene Ontology (GO) analysis based on predicted target genes indicated that quercetin may regulate the NF-κB signaling pathway in Pg.LPS-stimulated macrophages (Additional file 1: Fig. S5A). Immunofluorescence experiment indicated that p-P65 was translocated to the nucleus in Pg.LPS ( +) + Quercetin (-) group, and exposed cytoplasmic localization of NF-κB in Pg.LPS (-) + Quercetin (-) group. However, quercetin treatment obstructed the nuclear translocation of p-P65 caused by Pg.LPS stimulation, indicating that quercetin reduced the nuclear translocation of Pg.LPS-induced p-P65 (Additional file 1: Fig. S5B). Western blot was used to detect the expression of p-P65 in the nucleus of these groups. Consistent with the immunofluorescence results, p-P65 expression in the nuclei of the Pg.LPS ( +) + Quercetin (-) group was significantly elevated compared to the Pg.LPS (-) + Quercetin (-) group, while quercetin treatment significantly reduced the increase in Pg.LPS-induced levels of p-P65 expression (Fig. 5 C-D). The findings from this research revealed that quercetin could potentially hinder the activation of the Pg.LPS-induced NF-κB signaling pathway through the reduction of nuclear translocation of p-P65.

Firstly, we effectively upregulated and downregulated the levels of miR-21a-5p in RAW264.7 cells using miR-21a-5p mimic, inhibitor, and their negative control (NC) (Additional file 1: Fig. S5C). miR-21a-5p mimic yielded significantly increased levels of pro-inflammatory markers, including IL-1β, IL-6, iNOS and TNF-α, at both gene and protein levels, compared to the mimic NC group, as demonstrated in Fig. 5E–G by qRT-PCR and western blot tests. These findings suggest that the overexpression of miR-21a-5p could strengthen the inflammatory response of macrophages. To investigate miR-21a-5p's role further, we transfected RAW264.7 cells with miR-21a-5p mimic. As shown in Fig.  5H, I, the transfection of miR-21a-5p mimic significantly increased the expression of p-P65 in the nucleus, resulting in the activation of the NF-κB signaling pathway.

#### PDCD4 was a downstream target of miR-21a-5p and further regulated inflammatory responses of macrophages through the NF-κB signaling pathway

**Fig. 6:**
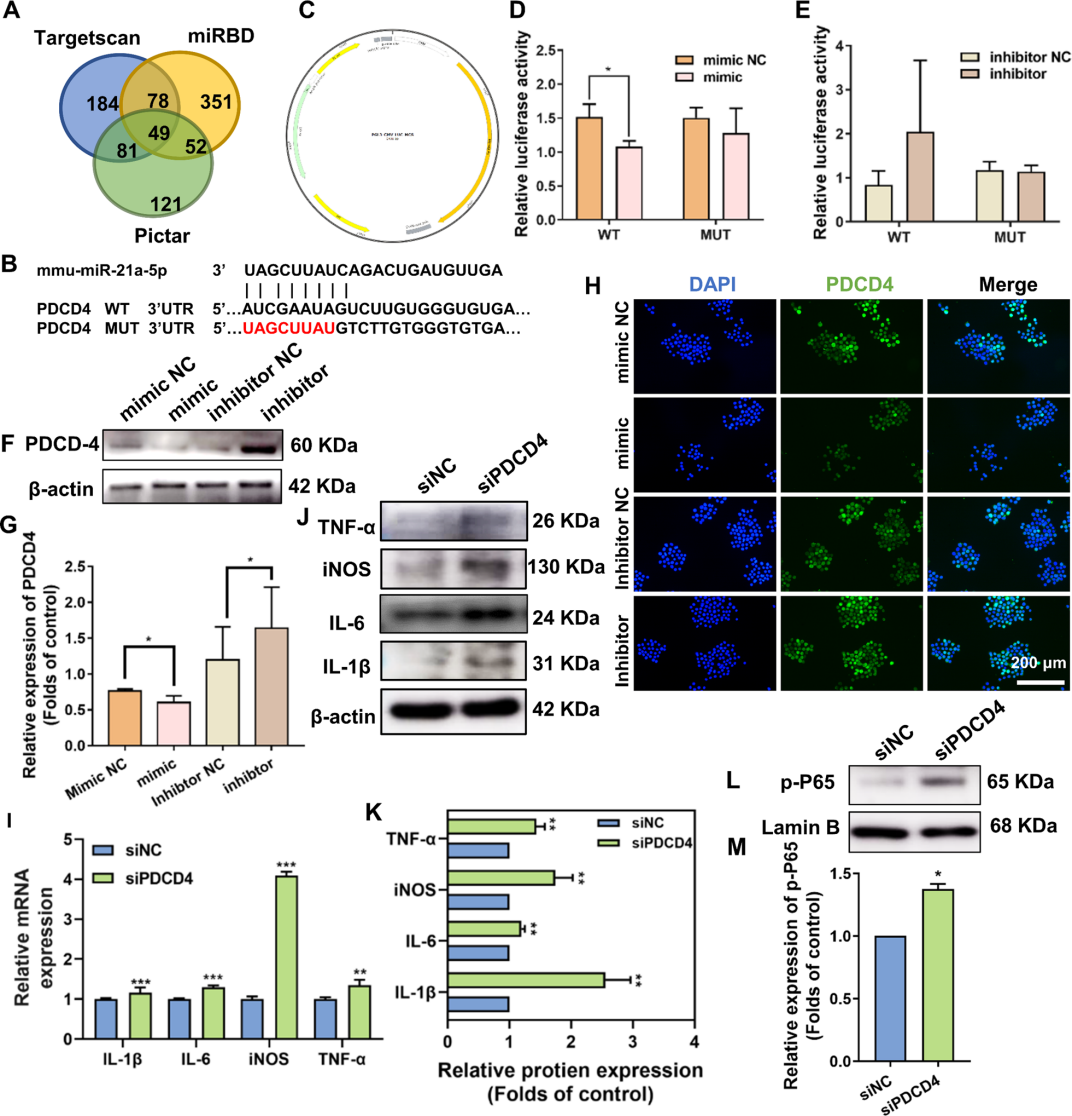
PDCD4 was a downstream target of miR-21a-5p and further regulated inflammatory responses of macrophages through the NF-κB signaling pathway. **A** Venn diagram of the overlapping target genes presented in Pg.LPS (-) + Quercetin (-) and Pg.LPS ( +) + Quercetin (-) groups. **B** The target sites of miR-21a-5p in the PDCD4 sequence predicted by bioinformatic software. **C** The construction profile of the pLUC-PDCD4 vector, which contained the miR-21a-5p target sites in the PDCD4 3′-UTR. **D**, **E** The results of dual-luciferase reporter assay after the co-transfection of the miR-21a-5p mimic, inhibitor, and their NC with PDCD4-WT-3′-UTR or PDCD4- mutant-type (MUT)-3′-UTR. **F**–**H** Western blot and immunofluorescence staining of the target protein PDCD4 in RAW264.7 cells after the transfection of the miR-21a-5p mimic, inhibitor, and their NC. **I–K** Effect of quercetin on the expression of inflammatory genes and proteins (IL-1β, IL-6, iNOS and TNF-α) through qRT-PCR and western blot in macrophages transfected with siNC and siPDCD4. **L,** **M** Western blot and its quantitative analysis of p-P65 following transfection of macrophages with siNC and siPDCD4. (^*^p < 0.05, ^**^p < *0.01 and* ^***^p < *0.001; Data are represented as the mean* ± *SEM, n* = *3)*

As non-coding single-stranded RNA, miRNAs can regulate mRNA by interacting with complementary base sequences of mRNA. We cross-referenced among three miRNA target prediction sites (Targets can, mired, and Pictar) to predict the potential targets of miR-21a-5p. We identified 49 potential targets of miR-21a-5p, of which PDCD4 was a well-documented downstream target of miR-21a-5p as reported in the literature (Fig. 6A) [32]. Therefore, we performed qRT-RCR, western blot and immunofluorescence staining experiments, and then found that PDCD4 expression decreased after adding Pg.LPS and subsequently increased after adding quercetin (Additional file 1: Fig. S5D–G). Furthermore, we choose the most effective siPDCD4 (siPDCD4-3) by using western blot (Additional file 1: Fig. S5H, I).

To determine whether miR-21a-5p targets the PDCD4 protein, we predicted the target site of miR-21a-5p within the PDCD4 sequence using bioinformatics software and designed the corresponding plasmids (Fig. 6B, C). To determine whether miR-21a-5p could target 3’-UTR of PDCD4, the luciferase reporter assay was carried out. The results showed that the overexpression of miR-21a-5p could remarkably attenuate the luciferase activity of the PDCD4 wild-type (WT) 3′-UTR (Fig. 6D, E). Subsequently, western blot and immunofluorescence staining experiments further confirmed that the up-regulation of miR-21a-5p inhibited the protein expression of PDCD4 in RAW264.7 cells, and vice versa (Fig. 6F–H). The outcomes of this study provided evidence that miR-21a-5p specifically targeted the PDCD4, leading to a decline in the PDCD4 expression.

To explore the role of PDCD4 in quercetin-mediated regulation of macrophage inflammatory response, qRT-PCR and western blot analyses were conducted, revealing that the suppression of PDCD4 expression could increase the expression of inflammatory genes and proteins as well as activate the NF-κB pathway (Fig. 6I–M).

The findings of this study supported the notion that miR-21a-5p specifically targeted the PDCD4 protein and downregulated PDCD4 expression, while the decline in PDCD4 expression promoted the inflammatory response and the activation of NF-κB pathway in RAW264.7 cells.

### *Quercetin performed indirect pro-osteo-/angiogenic effects *via* miR-21a-5p*

**Fig. 7:**
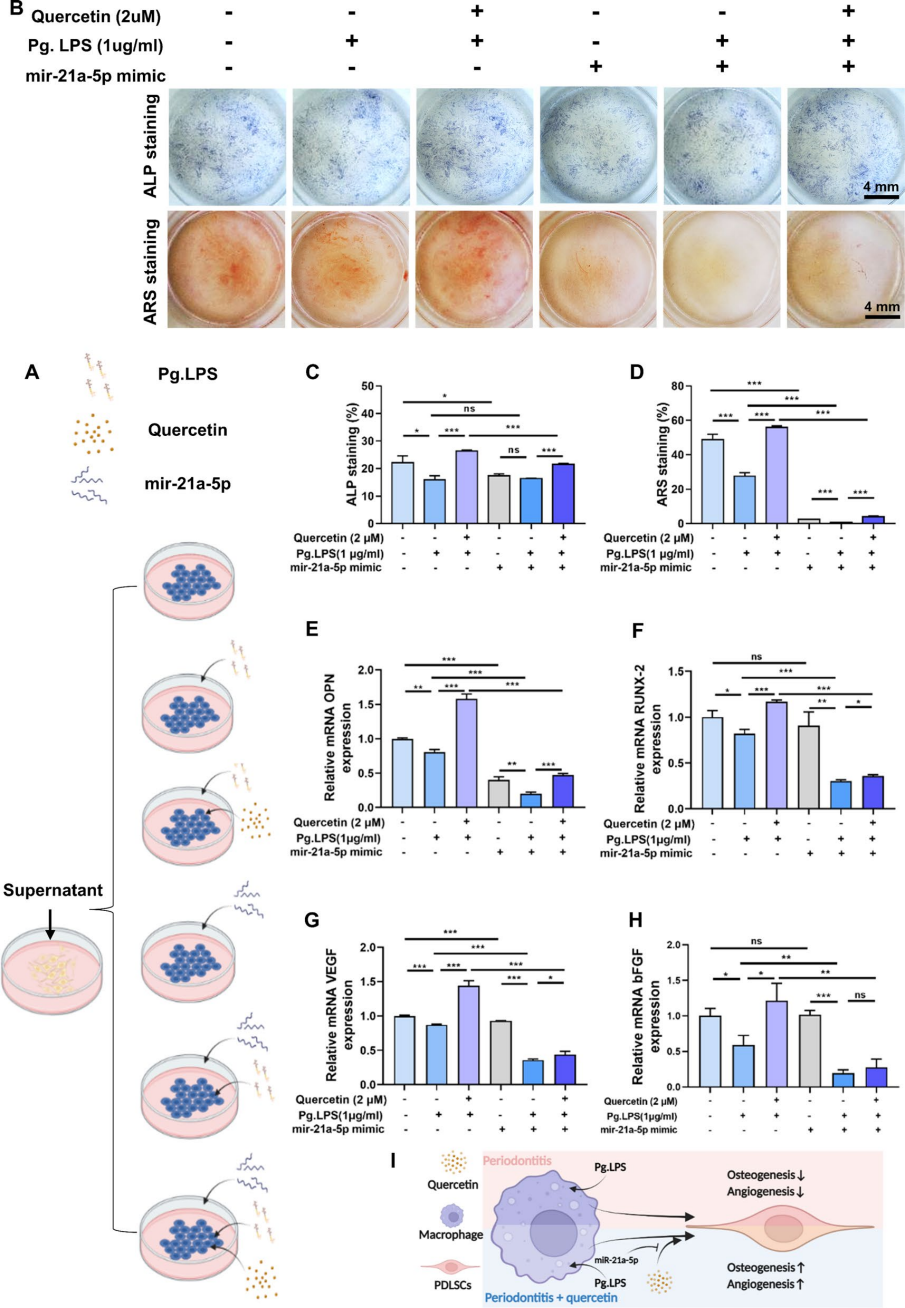
Quercetin performed indirect pro-osteo-/angiogenic effects via miR-21a-5p. **A** Schematic illustration of the co-culture experiment. **B**–**D** ALP staining, ARS staining and their quantitative results of PDLSCs cultured with the CM collected from macrophages for 7 and 21 days. **E**–**H** The expression of osteogenic-related (OPN and RUNX-2) and angiogenic-related (VEGF and bFGF) genes of PDLSCs cultured with the CM collected from macrophages through qRT-PCR. **I** Schematic of the pharmacological mechanism of quercetin and miR-21a-5p on osteoimmunomodulation between macrophages and PDLSCs under periodontitis microenvironment. (ns, no significant difference; ^*^p < 0.05, ^**^p < *0.01 and* ^***^p < *0.001; Data are represented as the mean* ± *SEM, n* = *3)*

To confirm whether the osteoimmunomodulatory effect of macrophages treated with quercetin through miR-21a-5p, the condition medium (CM) was collected from macrophages cultured with or without quercetin under inflammatory microenvironment pre-treated with miR-21a-5p mimic or mimic NC as shown in Fig. 7A. The CM was used to culture PDLSCs to evaluate their osteo-/angiogenic capacity via ALP staining, ARS staining as well as qRT-PCR. The results of ALP staining, ARS staining and their quantitative analyses of PDLSCs shown in Fig. 7B–D demonstrated that the treatment of quercetin upregulated the osteogenic differentiation of PDLSCs which had been weakened by pre-treatment with Pg.LPS. Additionally, except no significant difference observed between treatments Quercetin (2 μM) (-) + Pg.LPS (1 μg/mL) ( +) + miR-21a-5p mimic (-) and Quercetin (2 μM) (-) + Pg.LPS (1 μg/mL) ( +) + miR-21a-5p mimic ( +) in the ALP experiment, the osteogenic ability of PDLSCs was weaker under miR-21a-5p mimic treated conditions compared to those treated with miR-21a-5p mimic NC. Furthermore, aside from no significant difference shown in OPN gene (Quercetin (2 μM) (-) + Pg.LPS (1 μg/mL) (-) + miR-21a-5p mimic (-) vs Quercetin (2 μM) (-) + Pg.LPS (1 μg/mL) (-) + miR-21a-5p mimic ( +)) and bFGF gene (Quercetin (2 μM) (-) + Pg.LPS (1 μg/mL) (-) + miR-21a-5p mimic (-) vs Quercetin (2 μM) (-) + Pg.LPS (1 μg/mL) (-) + miR-21a-5p mimic ( +) and Quercetin (2 μM) (-) + Pg.LPS (1 μg/mL) ( +) + miR-21a-5p mimic ( +) vs Quercetin (2 μM) ( +) + Pg.LPS (1 μg/mL) ( +) + miR-21a-5p mimic ( +)), the expression trends of the osteogenic (OPN and Runx-2) and angiogenic (VEGF and bFGF) genes were generally congruent with those observed in ARS staining (Fig. 7E–H). Taken together, the above findings suggested that the osteoimmunomodulation of macrophages cultured with quercetin had a positive impact on osteo-/angiogenic differentiation of PDLSCs, while the overexpression of miR-21a-5p could block the quercetin’s indirect pro-osteo-/angiogenic effects (Fig. 7I).

### *Quercetin provided the protective effect in periodontitis *via* miR-21a-5p*

**Fig 8.**
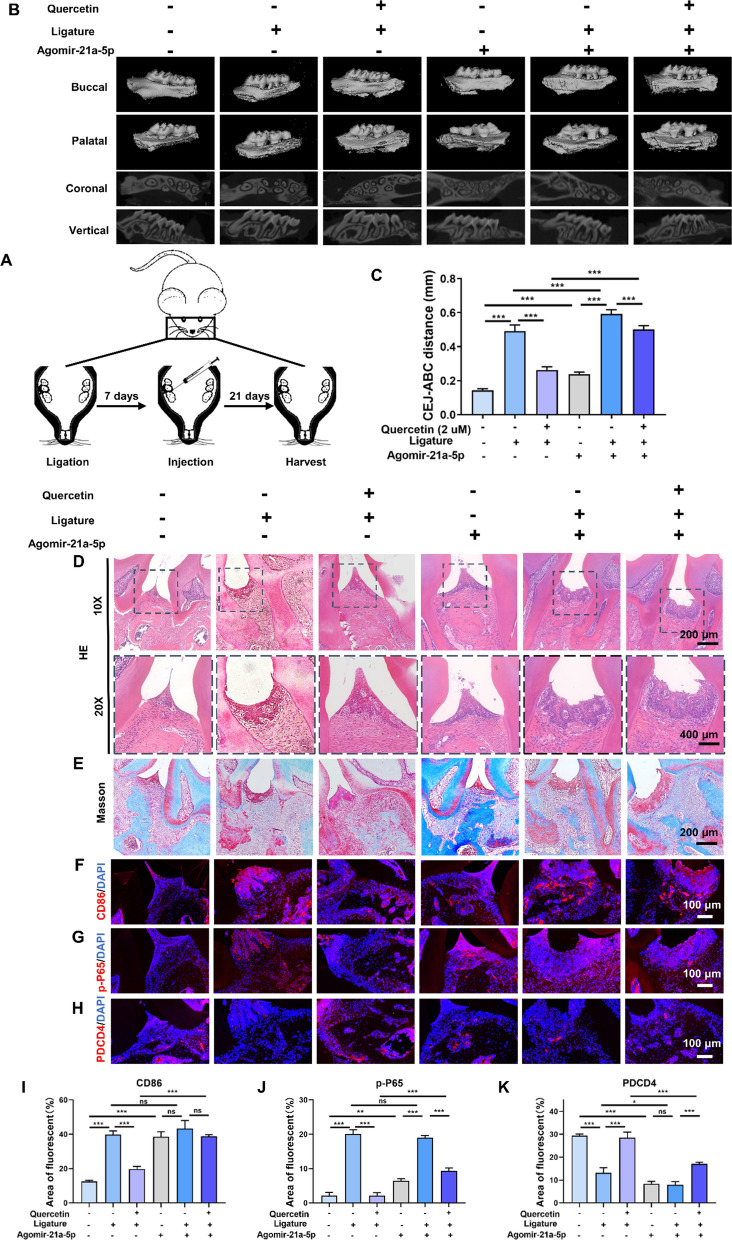
Quercetin provided the protective effect in periodontitis via miR-21a-5p. **A** Schematic illustration of the animal experiment. **B** The 3D reconstruction and 2D cross-section images through Micro-CT analysis. **C** The results of CEJ-ABC distance analysis. **D**–**H** H&E, Masson and immunofluorescence staining of CD86, p-P65 and PDCD4. **I**–**K** Quantitative analysis of the fluorescent area of CD86, p-P65 and PDCD4 according to Fig. 8**F**–**H**. (ns, no significant difference; ^*^p < 0.05, ^**^p < 0.01, ^***^p < *0.001; Data are represented as the mean* ± *SEM, n* = *3)*

To assess the immunoregulatory effect of quercetin in periodontitis and determine its miR-21a-5p dependence, C57BL/6 mice were first ligatured to establish an experimental periodontitis model and then were injected with either agomiR-21a-5p or its scrambled control in the absence or presence of quercetin as shown in Fig. 8A. Micro-CT analysis demonstrated that quercetin could obviously inhibit the bone resorption of periodontitis and substantially decrease the distance of cemento⁃enamel junction-alveolar bone crest (CEJ-ABC), whereas treatment with agomiR-21a-5p could partially inhibit the treatment effects of quercetin (Fig. 8B, C). H&E and Masson staining revealed that the quercetin-treated group reduced macrophage infiltration versus ligation group, while the addition of agomiR-21a-5p strengthened effects including increasing the number of inflammatory cells as well as blocking the protective effect of quercetin in inflammatory bone absorption (Fig. 8D, E).

To further investigate the in vivo immunomodulatory activity of the quercetin and miR-21a-5p, immunofluorescence staining of CD86, p-P65 and its quantitative analysis were performed (Fig. 8F-G, I-J). The CD86 and p-P65 protein level remarkably increased in the Quercetin (-) + ligation ( +) + agomiR-21a-5p (-) group and downregulated in Quercetin ( +) + ligation ( +) + agomiR-21a-5p (-) group, indicating the treatment of quercetin could effectively downregulate inflammatory response and inhibit the activation of NF-κB pathway. Furthermore, treatment with agomiR-21a-5p increased the expression of these two proteins, which could not be reduced by quercetin injection. However, there was no significant difference observed between the group treated with Quercetin ( +) + ligation ( +) + agomiR-21a-5p (-) and the one treated with Quercetin ( +) + ligation ( +) + agomiR-21a-5p ( +), which may be because the ligation procedure has already resulted in a significant expression of local inflammatory markers and the addition of agomiR-21a-5p could not be unable to further enhance it. This discovery suggested that miR-21a-5p could activate inflammatory status and block the anti-inflammatory capacity of quercetin. Moreover, PDCD4, as the downstream target gene of miR-21a-5p, was observed to decrease in response to the inflammatory environment and the high expression of miR-21a-5p as well as increase using quercetin, while the addition of agomiR-21a-5p could partially block the upregulatory effect of quercetin on PDCD4 expression (Fig. 8H, K). In summary, our results showed that quercetin possessed a superior in vivo anti-inflammatory effect by inhibiting the NF-κB pathway, while this function could be blocked by the high expression of miR-21a-5p.

### In vitro* evaluation of MBG's influence on quercetin's biological effects*

To evaluate whether delivering quercetin by MBG would alter the biological effect of quercetin, we assessed the effects of MBG, Quercetin, and Quercetin/MBG on the osteo-/angiogenic differentiation of PDLSCs and the inflammatory response of macrophages under periodontitis microenvironment. The study evaluated the osteo-/angiogenic differentiation of PDLSCs through ALP staining and qRT-PCR. Additional file 1: Fig. S6A, B showed that both the MBG and Quercetin groups were able to increase ALP activity, which had been inhibited by Pg.LPS. Moreover, the Quercetin/MBG group showed better efficacy than either of them individually. The qRT-PCR results were consistent with the ALP staining trend, except that VEGF gene expression was increased only in the Quercetin/MBG group (Additional file 1: Fig. S6C–F). The inflammatory response of macrophages was assessed using qRT-PCR. The findings suggested that MBG did not have an impact on the expression of inflammatory genes (IL-1β, IL-6, iNOS and TNF-α) that were activated in the periodontitis microenvironment. However, both Quercetin and Quercetin/MBG significantly down-regulated inflammatory genes, while there was no significant difference between the two groups (Additional file 1: Fig. S6G–J).

The study above demonstrated that quercetin exerted immunomodulatory effects through a key miRNA (miR-21a-5p) in macrophages. To evaluate whether MBG could affect the immunomodulatory mechanism of quercetin, qRT-PCR was performed. The result indicatde that MBG did not affect the expression of miR -21a-5p. Moreover, both the Quercetin and the Quercetin/MBG groups significantly suppressed the expression of miR-21a-5p, which was up-regulated by Pg.LPS, while there was no significant difference between the two groups (Additional file 1: Fig. S6K).

In summary, MBG could promote osteo-/angiogenesis without affecting the inflammatory response, while the addition of quercetin could further enhance osteo-/angiogenesis of PDLSCs and confer immunomodulatory properties to MBG (Additional file 1: Fig. S6L). Furthermore, our findings also indicated that the use of MBG did not interfere the immunomodulatory mechanism of quercetin.

## Discussion

Impaired osteo-/angiogenesis, excessive inflammatory activation, and subsequent disruption of osteoimmune homeostasis are the main reasons for the difficult healing of alveolar bone defects in periodontitis [4]. However, current treatments for periodontitis do not satisfactorily regenerate alveolar bone in periodontitis. Quercetin, as a natural flavonoid drug, has attracted wide attention in the treatment of inflammatory diseases due to its excellent regenerative and immunomodulatory abilities [33, 34]. The clinical application of this drug is limited in treating alveolar bone defects with periodontitis due to its poor water solubility and low bioavailability [35]. Recently, controlled release drug delivery systems were a promising strategy for treating periodontitis, which could control drug release as well as provide higher efficacy and fewer side effects [36]. Previous research has already reported that MBG could effectively load the flavonoid for the treatment of periodontitis [37]. Therefore, in this study, we produced a quercetin sustained-release system—Quercetin/MBG, which needed only one implant to be placed at the same time as surgery to achieve long-term effective sustained release of quercetin in the local area of alveolar bone defects with periodontitis. Moreover, our in vivo experiments showed that Quercetin/MBG treatment outperformed pure MBG treatment in both regenerating alveolar bone and managing osteoimmune microenvironment in periodontitis. Based on the aforementioned findings, it could be suggested that the use of a sustained-release system containing quercetin could be a novel approach towards regenerating alveolar bone in periodontitis.

Recent studies on periodontitis have shown that dysbiosis of local microbial communities may trigger local inflammation, whereas excessive activation of the host immune response can amplify inflammation, worsen tissue damage, and eventually cause irreversible periodontitis [38]. Therefore, remodeling the periodontal immune microenvironment is crucial for treating periodontitis. The periodontal immune microenvironment involves a variety of host cells, including MSCs and various immune cells. MSCs, especially PDLSCs, play a key role in the repair and regeneration of periodontal bone defects in periodontitis [39]. Interestingly, PDLSCs not only could directly aided in regenerating alveolar bone but also could excrete inflammatory substances that exacerbate the inflammatory microenvironment of periodontitis [40, 41]. High expression of inflammatory factors such as IL-6 and TNF-α by PDLSCs can stimulate downstream production of matrix metalloproteinases, leading to pathological degradation of periodontal extracellular matrix and the accelerated rupture of inflamed periodontal supporting tissues [42]. This study affirmed that quercetin possessed the ability to restore osteo-/angiogenic differentiation capacity as well as inhibit the pro-inflammatory factors’ production including IL-6 and TNF- α in inflammatory PDLSCs, which was beneficial for alveolar bone regeneration in periodontitis.

Macrophages are an important subset of immune cells in the development of periodontitis, which play roles in phagocytosis and immune regulation. Current evidence suggested that the proportion of pro-inflammatory M1 phenotypes was positively correlated with the progression of periodontal inflammatory activity, which could lead to sustained inflammation and tissue damage through the secretion of pro-inflammatory cytokines, such as IL-1β, IL-6, iNOS, TNF- α, etc. [43–45]. The study by Liu Y et al. showed that aspirin inhibited the activation of M1 macrophages, thereby significantly improving the regeneration of alveolar bone [46]. Thus, to control periodontitis, it is an effective strategy to inhibit the continuous activation of M1 macrophages. Our findings in vitro investigation demonstrated that quercetin effectively suppressed macrophage M1 polarization. Besides, macrophages could indirectly influence the behavior and function of PDLSCs via the secretion of paracrine factors, thereby affecting periodontal repair [47]. Thus, the importance of macrophages in osteoimmunomodulation during bone regeneration cannot be overlooked [48]. CM derived from M1 macrophages could significantly inhibit osteo-/angiogenic differentiation by releasing pro-inflammatory factors [49–51]. In our study, we revealed that the CM collected from M1 macrophages induced by Pg.LPS indeed could inhibit osteo-/angiogenesis of PDLSCs as well as the application of quercetin reversed the above inhibitory effects, providing an optimal osteoimmunomodulatory microenvironment for PDLSCs in vitro and leading to immune-enhanced osteo-/angiogenesis.

miRNA, due to its critical function in post-transcriptional gene modification, as a non-coding endogenous RNA, has gained tremendous attention in clinical applications such as inflammation therapy, tumor treatment etc. [52]. Studies have reported that miR-21 were involved in the periodontitis and the deficiency of miR-21 could alleviate alveolar bone loss in the experimental periodontitis consistent with our results [53, 54]. Furthermore, there was mounting evidence to suggest that the NF-κB signaling pathway played a crucial role in the development of periodontitis as well as inhibiting NF-κB signaling could provide protective and immunomodulatory effects against periodontitis [9, 55]. Previous findings have demonstrated that the NF-κB signaling could be upregulated by miR-21 [56, 57]. Moreover, the function of PDCD4 as a recognized target for miR-21 in regulating NF-κB signaling pathways has been reported in some literatures to be inhibitory [56, 58]. Consistent with our study, the overexpression of miR-21a-5p as well as the knockdown of PDCD4 significantly activated inflammatory reactions and the NF-κB signaling pathway in macrophages, while the quercetin could downregulate the miR-21a-5p expression, further decrease the downstream target PDCD4 and ultimately inhibit NF-κB signaling pathway activation. Additionally, we have reported for the first time that overexpression of miR-21 not only could generate an immune microenvironment that is unfavorable for bone regeneration but also could hinder the optimal osteoimmune microenvironment created by quercetin through macrophages in vitroand vivo. Moreover, we further demonstrated that the combination of the MBG delivery system and quercetin complemented each other: (1) MBG could assist quercetin to further enhance the osteo-/angiogenic differentiation of PDLSCs in the periodontal microenvironment; (2) The addition of quercetin could provide MBG with immunomodulatory properties, without interfering with quercetin's own excellent immunomodulatory properties.

Although the effect of Quercetin /MBG nano-delivery system on periodontal bone regeneration has been evaluated in a rat alveolar bone defect model with periodontitis, rodent models cannot fully reflect the real human disease situation and meet the needs of clinical translation of biomaterials [59]. Therefore, in the future study, we plan to introduce large animals to further evaluate the therapeutic effect of the quercetin nano-delivery system in regenerating periodontal bone defects under periodontitis.

## Conclusion

**Fig. 9:**
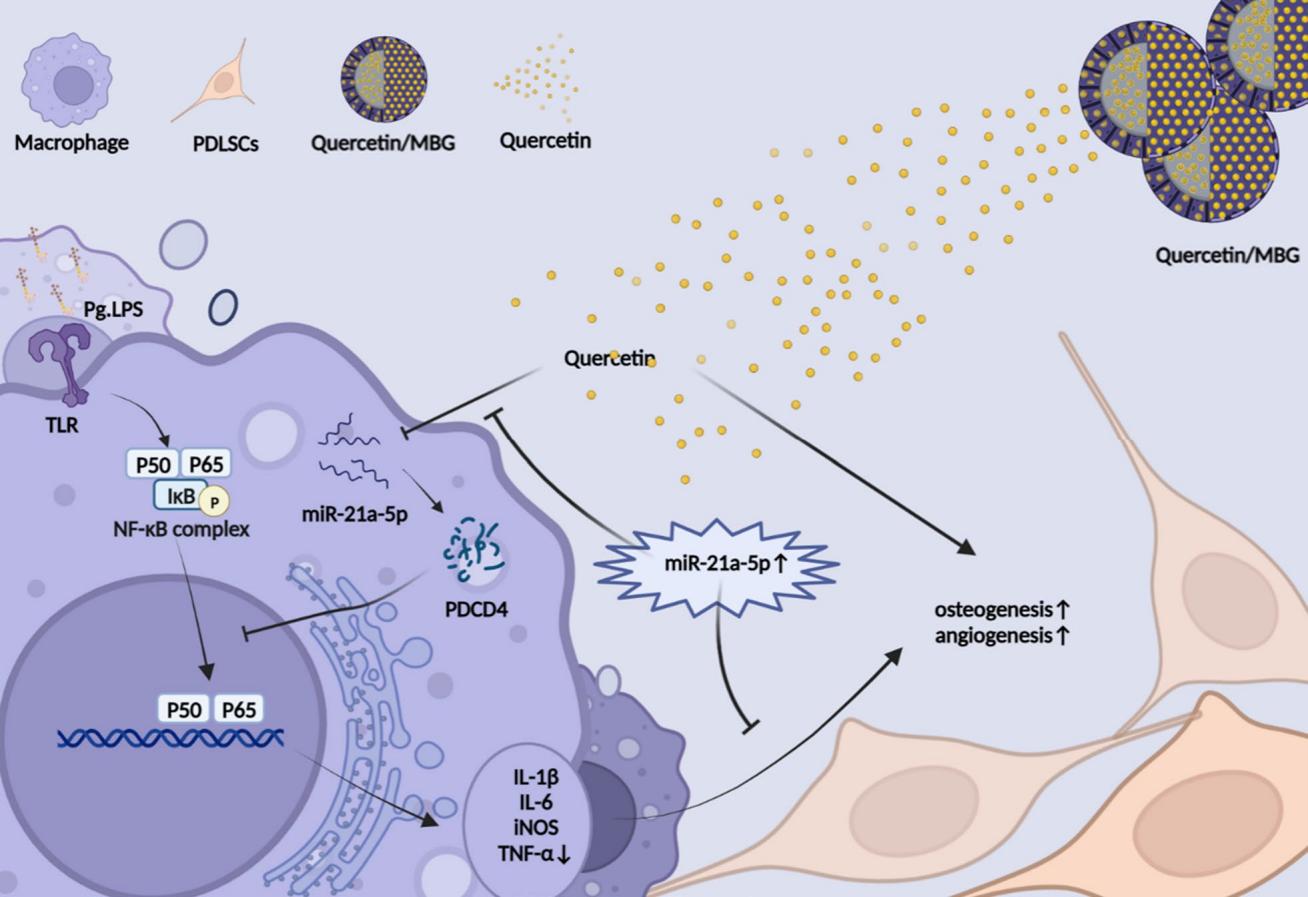
Schematic of the cellular mechanism of quercetin regulating macrophages and PDLSCs to promote alveolar bone regeneration in periodontitis

In summary, we certified that local delivery of quercetin could effectively promote vascularized bone regeneration and remodeling the osteoimmune microenvironment in alveolar bone defects with periodontitis. Besides, we also clearly clarified the pharmacological mechanisms that quercetin could restore the osteo-/angiogenic differentiation of PDLSCs, inhibit the M1 polarization of macrophages via the miR-21a-5p/PDCD4/NF-κB signaling axis and optimize the osteoimmune microenvironment via macrophages. Thus, this study reveals that nano-delivery system based on quercetin shows promise as an effective strategy for the treatment of alveolar bone defects with periodontitis (Fig. 9).

### Supplementary Information

**Additional file 1: Table S1.** qRT-PCR primer sequences used in this study. **Table S2.** qRT-PCR primer sequences used in this study. **Fig S1.** (A-B) The H & E and Masson staining of samples collected in 8 weeks. **Fig S2.** (A-B) Proliferation results of PDLSCs incubated with quercetin for 1,4 and 7 days in both physiological and periodontitis environment by using CCK-8 assay. (C) Live/Dead staining of PDLSCs on day 1 under the stimulation of quercetin under periodontitis environment. (#p < 0.05, ##p < 0.01 and ###p < 0.001 compared to Pg.LPS (-) + Quercetin (-); *p < 0.05, **p < 0.01 and ***p < 0.001 compared to Pg.LPS (+) + Quercetin (-); Data re represented as the mean ± SEM, n=3). **Fig S3.** (A-B) The protein level of PDCD4 in PDLSCs incubated with quercetin for 7 days under periodontitis environment by western blot. (#p < 0.05, ##p < 0.01 and ###p < 0.001 compared to Pg.LPS (-) +Quercetin (-); *p < 0.05, **p < 0.01 and ***p < 0.001 compared to Pg.LPS (+) +Quercetin (-); Data re represented as the mean ± SEM, n=3). **Fig S4.** (A-C) The expression of inflammation-related genes and protein of PDLSCs under periodontitis microenvironment after incubation with quercetin for 1 day. (#p < 0.05, ##p < 0.01 and ###p < 0.001 compared to Pg.LPS (-) +Quercetin (-); *p < 0.05, **p < 0.01 and ***p < 0.001 compared to Pg.LPS (+) +Quercetin (-); Data re represented as the mean ± SEM, n=3). **Fig S5.** (A) The Go enrichment analysis of Pg.LPS（+） + Quercetin（-） vs Pg.LPS（+） + Quercetin（+） group. (B)Immunofluorescence images of the p-P65 protein expression in macrophages. (C) The miR-21a-5p expression in RAW264.7 transfected by mir-21a-5p mimic NC, mimic, inhibitor NC and inhibitor. (D-G) qRT-PCR, western blot and immunofluorescence staining of the PDCD4 among Pg.LPS (-) +Quercetin (-), Pg.LPS (+) +Quercetin (-) and Pg.LPS (+) +Quercetin (+) groups. (H-I) The protein level of PDCD4 in siNC, siPDCD4-1-, siPDCD4-2- or siPDCD4-3-transfected macrophages determined by western blot. (In Fig. S5 D-F, #p < 0.05, ##p < 0.01 and ###p < 0.001 compared to Pg.LPS (-) + Quercetin (-) group and *p < 0.05, **p < 0.01 and ***p < 0.001 compared to Pg.LPS (+) + Quercetin (-) group. In Fig. S5 C and Fig. S6 I, *p < 0.05, **p < 0.01 and ***p < 0.001; Data are represented as the mean ± SEM, n=3). **Fig S6.** (A-B) ALP staining and its quantitative results after incubation of PDLSCs with MBG, Quercetin and Quercetin/MBG for 7 days under periodontitis microenvironment. (C-F) Effect of MBG, Quercetin and Quercetin/MBG on the expression of osteogenic-related genes (OPN and RUNX-2) and angiogenic-related genes (VEGF and bFGF) of PDLSCs under periodontitis microenvironment through qRT-PCR. (G-J) Effect of MBG, Quercetin and Quercetin/MBG on the expression of inflammatory genes (IL-1β, IL-6, iNOS and TNF-α) of RAW264.7 under periodontitis microenvironment through qRT-PCR. (K) Effect of MBG, Quercetin and Quercetin/MBG on the expression of miR-21a-5p of RAW264.7 under periodontitis microenvironment through qRT-PCR. (L) Schematic of Quercetin/MBG on PDLSCs and RAW264.7 under periodontitis microenvironment. (ns, no significant difference; *p < 0.05, **p < 0.01 and ***p < 0.001; Data are represented as the mean ± SEM, n=3).

## Data Availability

The datasets used and/or analyzed during the current study are available from the corresponding author on reasonable request.
