# Supplementary material for: Quercetin-loaded mesoporous nano-delivery system remodels osteoimmune microenvironment to regenerate alveolar bone in periodontitis via the miR-21a-5p/PDCD4/NF-κB pathway
Source: J Nanobiotechnology. 2024 Mar 6;22:94. doi: 10.1186/s12951-024-02352-4 (PMC10918894; doi:10.1186/s12951-024-02352-4)
Supplement: Supplementary file 1 — Additional file 1: Table S1. qRT-PCR primer sequences used in this study. Table S2. qRT-PCR primer sequences used in this study. Fig S1. (A-B) The H & E and Masson staining of samples collected in 8 weeks. Fig S2. (A-B) Proliferation results of PDLSCs incubated with quercetin for 1,4 and 7 days in both physiological and periodontitis environment by using CCK-8 assay. (C) Live/Dead staining of PDLSCs on day 1 under the stimulation of quercetin under periodontitis environment. (#p < 0.05, ##p < 0.01 and ###p < 0.001 compared to Pg.LPS (-) + Quercetin (-); *p < 0.05, **p < 0.01 and ***p < 0.001 compared to Pg.LPS (+) + Quercetin (-); Data re represented as the mean ± SEM, n=3). Fig S3. (A-B) The protein level of PDCD4 in PDLSCs incubated with quercetin for 7 days under periodontitis environment by western blot. (#p < 0.05, ##p < 0.01 and ###p < 0.001 compared to Pg.LPS (-) +Quercetin (-); *p < 0.05, **p < 0.01 and ***p < 0.001 compared to Pg.LPS (+) +Quercetin (-); Data re represented as the mean ± SEM, n=3). Fig S4. (A-C) The expression of inflammation-related genes and protein of PDLSCs under periodontitis microenvironment after incubation with quercetin for 1 day. (#p < 0.05, ##p < 0.01 and ###p < 0.001 compared to Pg.LPS (-) +Quercetin (-); *p < 0.05, **p < 0.01 and ***p < 0.001 compared to Pg.LPS (+) +Quercetin (-); Data re represented as the mean ± SEM, n=3). Fig S5. (A) The Go enrichment analysis of Pg.LPS（+） + Quercetin（-） vs Pg.LPS（+） + Quercetin（+） group. (B)Immunofluorescence images of the p-P65 protein expression in macrophages. (C) The miR-21a-5p expression in RAW264.7 transfected by mir-21a-5p mimic NC, mimic, inhibitor NC and inhibitor. (D-G) qRT-PCR, western blot and immunofluorescence staining of the PDCD4 among Pg.LPS (-) +Quercetin (-), Pg.LPS (+) +Quercetin (-) and Pg.LPS (+) +Quercetin (+) groups. (H-I) The protein level of PDCD4 in siNC, siPDCD4-1-, siPDCD4-2- or siPDCD4-3-transfected macrophages determined by western blot. (In Fig. S5 D-F, #p < 0 [file 12951_2024_2352_MOESM1_ESM.docx]

**Supporting Information for**

**Quercetin-loaded mesoporous nano-delivery system remodels osteoimmune microenvironment to regenerate alveolar bone in periodontitis via the miR-21a-5p/PDCD4/NF-κB pathway**

*Shi-Yuan Yang^a, b, 1^, Yue Hu^a, b, 1^, Ran Zhao**^b,c^, Yu-Ning Zhou^a, b^, Yu Zhuang^b, d^, Yan Zhu^a, b^, Xiao-Li Ge**^b,c^, Ting-Wei Lu^b, d^, Kai-Li Lin^b, d, **^, Yuan-Jin Xu ^a, b, *^*

*^a^**Department of Oral Surgery,* *Shanghai Ninth People’s Hospital, Shanghai Jiao Tong University School of Medicine, Shanghai, China*

*^b^**College of Stomatology, Shanghai Jiao Tong University; National Center for Stomatology; National Clinical Research Center for Oral Diseases; Shanghai Key Laboratory of Stomatology; Shanghai Research Institute of Stomatology, Shanghai, China*

*^c^Department of Oral Mucosal Diseases, Shanghai Ninth People’s Hospital, Shanghai Jiao Tong University School of Medicine, Shanghai, China*

*^d^Department of Oral and Cranio-maxillofacial Surgery, Shanghai Ninth People’s Hospital, Shanghai Jiao Tong University School of Medicine, Shanghai, China*

*Peer review under responsibility of KeAi Communications Co., Ltd.*

*∗ Corresponding author. Shanghai Ninth People's Hospital, Shanghai Jiao Tong University School of Medicine, 639 Zhizaoju Road, Shanghai, 200011, China.*

*∗∗ Corresponding author. Shanghai Ninth People's Hospital, Shanghai Jiao Tong University School of Medicine, 639 Zhizaoju Road, Shanghai, 200011, China.*

*E-mail addresses:* ***Corresponding authors:*** *drxuyuanjin@126.com (Yuanjin Yuan); linkaili@sjtu.edu.cn & lklecnu@aliyun.com (Kaili Lin).*

***Other authors:*** *Yangshiyuan1996@sjtu.edu.cn (Shiyuan Yang); moonhu1992@163.com (Yue Hu); jjs3156@163.com (Ran Zhao); xiaoyao7958@163.com (Yuning Zhou); Zhuangyu951102@163.com (Yu Zhuang); k.atherine@sjtu.edu.cn (Yan Zhu); a1211301544@163.com (Xiaoli Ge); 541197081@sjtu.edu.cn (Tingwei Lu).*

**Supplementary Methods**

***Materials and regents***

Quercetin was obtained from Sigma Aldrich (MO, USA), while Lipopolysaccharide from P. gingivalis (Pg.LPS) was supplied by invivogen (CA, USA). Gibco (CA, USA) provided Dulbecco’s modified Eagle’s medium (DMEM), α-Minimum Essential Medium(α-MEM), fetal bovine serum (FBS), Trypsin-EDTA (0.25%), phosphate buffer saline (PBS) and penicillin/streptomycin(P/S). The following reagents were purchased from Macklin (Shanghai, China): Dexamethasone, β-Glycerol phosphate disodium salt and sodium ascorbate. Dojindo (Tokyo, Japan) supplied CCK-8 and calcein-AM/propidium iodide (PI) double staining kit. Bicinchoninic acid (BCA) protein kit, BCIP/NBT alkaline phosphatase (ALP) color development kit, 2-(4-Amidinophenyl)-6-indolecarbamidine dihydrochloride (DAPI), RIPA Lysis Buffer, Bovine Serum Albumin (BSA) as well as Nuclear and Cytoplasmic Protein Extraction Kit were obtained from Beyotime (Shanghai, China). 1% alizarin red staining (ARS, pH 4.2) was manufactured by Solarbio (Beijing, China). PAGE Gel Fast Preparation Kit, Protein Sample Loading Buffer, Protein Free Rapid Blocking Buffer, Omni-Flash™ Western Blot Rapid Transfer Buffer, TBS/Tween Buffer were obtained from epizyme Co., Ltd. (Shanghai, China). Immobilon Western HRP Substrate and polyvinylidene fluoride (PVDF) was purchased from Millipore (MA, UAS). TRIzol reagent, PrimerScript™ RT Master Mix kit, TB Green Premix Ex Taq™ kit and YFDye Phalloidin Conjugates were accommdated by Takara (Tokyo, Japan). Sangon Biotech Co., Ltd. (Shanghai, China) synthesized the primers miRNA and the first strand cDNA synthesis kit (Tailing Reaction) used in this study. RiboBio (Guangzhou, China) provided miR-21a-5p mimic, miR-21a-5p inhibitor, PDCD4 siRNA and their correspondingly NC as well as riboFECT CP transfection kit. pLUC-PDCD4- WT- 3′-UTR, pLUC-PDCD4- MUT-3′-UTR plasmid, agomir-21a-5p and agomir-21a-5p NC were synthetized by Genomeditec (Shanghai, China). Lipofectamine® 3000 was purchased from Thermo Fisher Scientific (MA, USA).

***In vivo verification of local sustained-release quercetin in a rat alveolar bone defect model with periodontitis***

**Preparation of the quercetin/MBG** **drug delivery system**: As previously reported, mesoporous bioactive glass (MBG) nanoparticles were prepared using a modified sol-gel method, which involved cetyltrimethylammonium bromide (CTAB) as a template reagent^1^. Briefly, the precursor solution for MBG nanoparticles was prepared using the following method: CTAB (3.3 mM) was dissolved in deionized water (DDW, 165 ml) and absolute ethanol (ETOH, 78 ml) with continuous stirring. The CTAB was fully dissolved before adding 25 wt% ammonia solution (3 ml), tetraethyl orthosilicate (TEOS, 3 ml), triethyl phosphate (TEP, 0.23 ml) and calcium nitrate tetrahydrate (CN, 0.64 g), with magnetic stirring intervals of 30 minutes. The resulting mixture was vigorously stirred at room temperature for 3 hours and the clear solution gradually became opaque due to the formation of a white precipitate. Before being dried at room temperature for 24 hours, the white precipitate was collected through filtration and rinsed with ETOH and DW. To remove the template, the precursor was sintered in air at 650°C (2°C/min) for 3 hours.

As previously reported, 300 mg of quercetin (dissolved in 6 mL of ETOH) was mixed with 80 mg of MBG and the mixture was stirred at 100 rpm for 24 hours in a rotary incubator^2^. Quercetin-loaded MBG (Quercetin/MBG) were isolated by centrifugation, and excess quercetin adsorbed on the outer surface was removed via washing with ETOH and water. The morphology and characteristics of MBG were determined by transmission electron microscope (TEM), X-ray diffraction (XRD) and fourier transform infrared spectroscopy (FTIR).

The loading capacity and encapsulation efficiency of quercetin/MBG were determined using UV-VIS spectrophotometry. Briefly, 1.0 mg of quercetin/MBG was suspended in 2.0 mL of ETOH and mixed using magnetic stirring at 37℃ for 24 hours, followed by centrifugation at 2000 rpm for 5 minutes. The supernatant's absorbance value was measured at 375 nm using a NanoDrop™ 8000 spectrophotometer (ND-8000-GL, Thermo Scientific). The absorbance of quercetin ethanol solution at a series of concentrations was also measured at 375 nm to create a reference curve. The loading capacity and encapsulation efficiency of the drug were then calculated using the following formula:

$$Loading capacity (mg/g)=\frac{Mass of quercetin loaded in MBG}{Mass of MBG}$$

$$Encapsulation efficiency \left( \% \right)=\frac{Mass of quercetin loaded in MBG}{Mass of quercetin in feed} \times100\%$$

The *in vitro* drug release study of quercetin was performed under a 100-rpm shaking condition by incubating of 10 mg of quercetin/MBG in 1 mL of artificial saliva. At preplanned time points, the sample was taken and centrifugated at 2000 rpm for 5 minutes. Subsequently, a half of supernatant was removed to determine the absorbance at 374 nm and replaced with an equal volume of artificial saliva. Various concentrations of quercetin dissolved in artificial saliva were used to construct a standard curve line by recording the absorbance at 374 nm. This standard curve line was created for use in calculating the cumulative release concentrations of quercetin.

***In vivo* assay of the therapeutical effect of quercetin/MBG delivery system:** The animal research was approved by the Independent Ethics Committee of Shanghai Ninth People's Hospital. It followed the National Institutes of Health guide for the care and use of laboratory animals (SH9H-2020-A469-1).

The experimental periodontitis model was induced in rats using ligation according to previously published methods ^[25, 26]^. Briefly, the rats were given intraperitoneal anesthesia, and ligation wires (with a diameter of 0.22 μm) were placed on the cervix of their first molars. Afte 3 weeks of ligation, the ligation wires were removed, and the periodontal area was completely cleaned of food debris, plaque, and calculus.

After the experimental periodontitis model was established, we created the alveolar bone defect with a radius and depth of 3 mm at the medial portion of the maxillary first molars using a dental drill. The prepared MBG and quercetin/MBG nanoparticles were immediately implanted into the bone defect, while the rats without any implant materials was set as the blank group. The animals were sacrificed at 1-, 4-, and 8-weeks post-surgery, and the alveolar bone was assessed through the utilization of Micro-CT scanning to measure bone regeneration. The tissues were then decalcified, embedded in paraffin wax, and sectioned for staining using hematoxylin and eosin (H&E), Masson and tissue immunofluorescence.

***Cell culture***

Periodontal ligament stem cells (PDLSCs) were isolated according to a previously described method^3^. Primary PDLSCs were maintained in α-MEM supplemented with 10% (v/v) FBS and 1% (v/v) P/S. Upon reaching 90% confluence, PDLSCs were passaged using trypsin.

The murine-derived macrophage RAW 264.7 cell line (Cell Bank of Chinese Academy of Sciences, China) was cultured in complete DMEM.

The cells mentioned above were all cultured at 37°C in an incubator with 5% CO_2_.

***Influence of quercetin on osteo-/angiogenesis activity of PDLSCs***

The PDLSCs were exposed to varying concentrations of quercetin (0.5, 1, 2, and 4 μM) in the absence or presence of Pg.LPS at a concentration of 1 μg/mL. PDLSCs were cultured for 1, 4, and 7 days, and their cell viability was evaluated using the CCK-8 test. On day 1, PDLSCs were stained using the calcein-AM/PI double staining kit, and fluorescence images were captured with an inverted microscope (Ti-S, Nikon, Japan).

After being treated for 4 and 7 days, BCIP/NBT ALP color development kit was employed to perform ALP staining. Samples collected on days 14 and 21 were subjected to ARS staining. Stereomicroscope (SMZ25, Nikon, Japan) was employed to capture the staining samples.

Gene expression in samples cultured for 7 days was evaluated for osteo-/angiogenic genes, while gene expression in samples cultured for 1 day was assessed for inflammatory genes, using qRT-PCR according to the manufacturer's protocols. The primers used in this section were shown in Table S1 and glyceraldehyde 3-phosphate dehydrogenase (GAPDH) was used as the internal reference.

Angiogenic protein expression was evaluated in samples cultured for 7 days using western blot analysis. The cells' total protein was lysed with RIPA Lysis Buffer, and the protein concentration was determined with the BCA protein kit. Proteins were separated using the PAGE Gel Fast Preparation Kit, transferred to PVDF membranes, and blocked with 5% BSA. The blocked PVDF membranes were incubated with vascular endothelial growth factor (VEGF, AF5131, Affinity Biosciences) and β-actin (#4967, CST) antibodies overnight at 4 ° C. Goat anti-Rabbit IgG (H+L)-HRP Antibody (abs20147, Absin) was later incubated for 1 hour at room temperature and developed in Amersham ImageQuant (Amersham, USA) using Immobilon Western HRP substrate.

To compare the expression of osteo-/angiogenic and inflammatory proteins among different groups of PDLSCs, cellular specimens collected on days 3 and 7 were incubated with primary antibodies overnight at 4°C, followed by incubation with Alexa Fluor 594-conjugated Donkey Anti-Mouse IgG H&L (ab150116, Abcam). Visualization of cytoskeleton and nuclei was achieved using Actin-Tracker Green-488 and DAPI, respectively. The primary antibodies used in this section were as follows: osteopontin (OPN, 1B20, NOVUS), VEGF (MA5-13182, Thermo Fisher Scientific) and interleukin-6 (IL-6, D5W4V, CST). The fluorescence images received above were photographed by upright microscope (DS-Ri2, Nikon, Japan).

***Influence of quercetin on inflammatory response of macrophages***

RAW264.7 cells were subjected to the same treatment as PDLSCs, as stated above. Cell viability was evaluated using the CCK-8 assay following 24 hours of culture, and the expression levels of inflammation-related genes were assessed via qRT-PCR as described above after 24 hours of culture. The primers used in this section were listed in Table 2. The supernatants of each culture were collected and used to assess the concentrations of interleukin-1β (IL-1β, abs520001, Absin), IL-6 (abs520004, Absin), tumor necrosis factor-α (TNF-α, abs520010, Absin) and nitric oxide (NO, S0021S, Beyotime) by using corresponding kit after cultured for 3 days.

***Regulation mechanism of quercetin on macrophages based on miRNA sequencing assay***

Subsequently, total RNA from RAW264.7 cells cultured in the Pg.LPS (+) + Quercetin (-) and Pg.LPS (+) + Quercetin (+) groups was extracted to perform a microarray assay by GMINIX (Shanghai, China). The miRNA first strand cDNA synthesis (Tailing Reaction) and TB Green Premix Ex Taq™ kit were applied to complete the qRT-PCR to verify the expression of miR-21a-5p according to the instructions guide. Endogenous U6 expression was used for normalization. Primer sequences were also described in Table S2.

The riboFECT CP transfection kit was used to transfect miR-21a-5p mimic, miR-21a-5p inhibitor, PDCD4 siRNA, and their NC into the RAW 264.7 cells. qRT-PCR as mentioned above was processed using to evaluate the transfection efficiency of miR-21a-5p mimic and inhibitor. To study the relationship between miR-21a-5p and PDCD4, a dual-luciferase reporter assay was performed by co-transfection of the miR-21a-5p mimic, miR-21a-5p inhibitor, or their NC with the pLUC-PDCD4-WT 3′-UTR or pLUC-PDCD4-MUT 3′-UTR plasmid using Lipofectamine^®^ 3000. And after 36 hours of transfection, the luciferase activity was measured by the microplate reader (Biotek, Synergy H1). Western blot and immunofluorescence were used to further confirm the expression of PDCD4 after macrophages treated with quercetin or transfect the miR-21a-5p mimic, miR-21a-5p inhibitor, or their NC.

Gene and protein levels of PDCD4, IL-1β, IL-6, iNOS, and TNF-α were determined by qRT-PCR and western blot analysis. The Nuclear and Cytoplasmic Protein Extraction Kit was used to extract nuclear proteins from cells after the indicated treatment. In addition, the activation of the NF-κB pathway was assessed using western blot and immunofluorescence analyses. The primary antibodies utilized in immunofluorescence and western blot were showed as follows: p-P65 (Ser536, CST), PDCD4 (D29C6, CST), IL-1β (D6D6T, CST), IL-6 (D5W4V, CST), iNOS (D6B6S, CST), TNF-α (26405-1-AP, Prointech), Lamin B (D9V6H, CST) and β-actin (20536-1-AP, Prointech). The western blot employed Goat anti-Rabbit IgG (H+L)-HRP Antibody (abs20147, Absin) and Goat anti-Mouse IgG (H+L)-HRP Antibody (abs20163, Absin). In addition, immunofluorescence utilized Alexa Fluor 594-conjugated Goat Anti-Rabbit IgG H&L (ab150080, abcam) and Alexa Fluor 488-conjugated Goat Anti-Rabbit IgG H&L (ab150077, abcam) as fluorescent secondary antibodies.

***The osteoimmunomodulatory function of macrophages cultured with quercetin***

To investigate the osteoimmunomodulatory effects of macrophages, we collected the supernatant from macrophages that were cultured in groups as follows for 72 hours as conditioned medium (CM) for further study: (1) Quercetin (2 μM) (-) + Pg.LPS (1 μg/ml) (-) + miR-21a-5p mimic (-); (2) Quercetin (2 μM) (-) + Pg.LPS (1 μg/ml) (+) + miR-21a-5p mimic (-); (3) Quercetin (2 μM) (+) + Pg.LPS (1 μg/ml) (+) + miR-21a-5p mimic (-); (4) Quercetin (2 μM) (-) + Pg.LPS (1 μg/ml) (-) + miR-21a-5p mimic (+); (5) Quercetin (2 μM) (-) + Pg.LPS (1 μg/ml) (+) + miR-21a-5p mimic (+);（6）Quercetin (2 μM) (+) + Pg.LPS (1 μg/ml) (+) + miR-21a-5p mimic (+).

The osteo-/angiogenesis of PDLSCs co-cultured with CM was measured by ALP staining, ARS staining and qRT-PCR analysis as previously mentioned.

***In vivo immunomodulatory function of quercetin in a mouse model with periodontitis***

Forty-eight 7-week-old male C57BL/6 mice were obtained to create the experimental periodontitis model and assigned into six groups of 8 mice each. After being anesthetized with 2% avertin (0.1 mL/10 g), 5-0 ligature wire was used to ligate the mesial and distal space around the second maxillary molar. Following a week of ligation, 10 µL of 2μM quercetin solution and PBS (every 2 days) as well as agomir-21a-5p and its NC (every 7 days) were injected into the central palatal submucoperiosteal region with a microsyringe, while the ligature wires were retained during treatment. The 6 groups were defined as follows: (1) Quercetin (-) + ligature (-) + agomir-21a-5p (-); (2) Quercetin (-) + ligature (+) + agomir-21a-5p (-); (3) Quercetin (+) + ligature (+) + agomir-21a-5p (-); (4) Quercetin (-) + ligature (-) + agomir-21a-5p (+); (5) Quercetin(-) + ligature (+) + agomir-21a-5p (+); (6) Quercetin (+) + ligature (+) + agomir-21a-5p (+). 3 weeks after injection, the mice were executed as well as the complete maxillary bone containing the maxillary teeth were collected and fixed in 4% PFA. After undergoing Micro-CT analysis, the fixed samples were decalcified and embedded for subsequent H&E, Masson and tissue immunofluorescence staining.

***In vitro evaluation of MBG's influence on quercetin's biological effects***

To investigate *in vitro* evaluation of MBG's influence on quercetin's biological effects, we first cultured MBG and quercetin/MBG in complete medium at 37 ° C for 72 hours, after which the supernatant was taken as the extract. Cellar experiments were performed as follows: (1) Control group; (2) Pg.LPS group; (3) Pg.LPS + MBG (extract) group; (4) Pg.LPS + Quercetin group; (5) Pg.LPS + Quercetin/MBG (extract) group. The osteo-/angiogenesis of PDLSCs was measured by ALP staining, qRT-PCR analysis as previously mentioned. The osteo-/angiogenesis of PDLSCs was measured by ALP staining, qRT-PCR analysis as previously mentioned. The inflammatory response of RAW264.7 was measured by qRT-PCR analysis as previously mentioned. The inflammatory mechanism of RAW264.7 was measured by qRT-PCR analysis as previously mentioned.

***Statistical analysis***

The data were analyzed using one-way ANOVA and presented as means ± standard deviations (SD). The experiments were repeated at least three times. GraphPad Prism 9.0 was used for statistical analyses.

**Supplementary Tables**

**Table S1 qRT-PCR primer sequences used in this study.**

| Gene | Full name | Primer | Sequences (5′–3′) |
| --- | --- | --- | --- |
| GAPDH | Glyceraldehyde 3-phosphate dehydrogenase | Forward  Reverse | GGCTGTTGTCATACTTCTCATGG |
|  |  |  | GGAGCGAGATCCCTCCAAAAT |
| OPN | **Osteopontin** | Forward  Reverse | CAGGTCTGCGAAACTTCTTAGAT |
|  |  |  | CTCCATTGACTCGAACGACTC |
| RUNX-2 | RUNX family transcription factor - 2 | Forward  Reverse | TCTCAGATCGTTGAACCTTGCTA |
|  |  |  | TGGTTACTGTCATGGCGGGTA |
| bFGF | Basic fibrobast growth factor | Forward  Reverse | GCGACCCTCACATCAAGCTA |
|  |  |  | AGCCAGGTAACGGTTAGCAC |
| VEGF | Vascular endothelial growth factor | Forward  Reverse | GTGAGGTTTGATCCGCATAATC |
|  |  |  | ATCGAGTACATCTTCAAGCCAT |
| IL-6 | Interleukin-6 | Forward  Reverse | GTGCCCATGCTACATTTGCC |
|  |  |  | TGCAATAACCACCCCTGACC |
| TNF-α | Tumor necrosis factor-α | Forward  Reverse | AGGAAGGCCTAAGGTCCACT |
|  |  |  | CACAGTGAAGTGCTGGCAAC |

**Table S2 qRT-PCR primer sequences used in this study.**

| **Gene** | | **Full name** | **Primer** | **Sequences (5′–3′)** |
| --- | --- | --- | --- | --- |
| **IL-1β** | Interleukin-1β | | Forward  Reverse | TGGAGAGTGTGGATCCCAAG |
|  |  |  |  | GGTGCTGATGTACCAGTTGG |
| **IL-6** | Interleukin-6 | | Forward  Reverse | ATAGTCCTTCCTACCCCAATTTCC |
|  |  |  |  | GATGAATTGGATGGTCTTGGTCC |
| **iNOS** | Inducible Nitric Oxide Synthase | | Forward  Reverse | CTGCTGGTGGTGACAAGCACATTT |
|  |  |  |  | ATGTCATGAGCAAAGGCGCAGAAC |
| **TNF-α** | Tumor necrosis factor-α | | Forward  Reverse | TGGGAGTAGACAAGGTACAACCC |
|  |  |  |  | CATCTTCTCAAAATTCGAGTGACAA |
| **β-actin** | β-actin | | Forward  Reverse | GGCTGTATTCCCCTCCATCG |
|  |  |  |  | CCAGTTGGTAACAATGCCATGT |
| **miR-21a-5p** |  | | Forward | GCGCGTAGCTTATCAGACTGA |
|  |  |  | Reverse | AGTGCAGGGTCCGAGGTATT |
| **U6** |  | | Forward  Reverse | CTCGCTTCGGCAGCACATATACT |
|  |  |  |  | ACGCTTCACGAATTTGCGTGTC |
| **miR-21a-5p** |  | | RT Primer | GTCGTATCCAGTGCAGGGTCCGAGG  TATTCGCACTGGATACGACTCAACA |
| **U6** |  | | RT Primer | AAAATATGGAACGCTTCACGAATTTG |

**
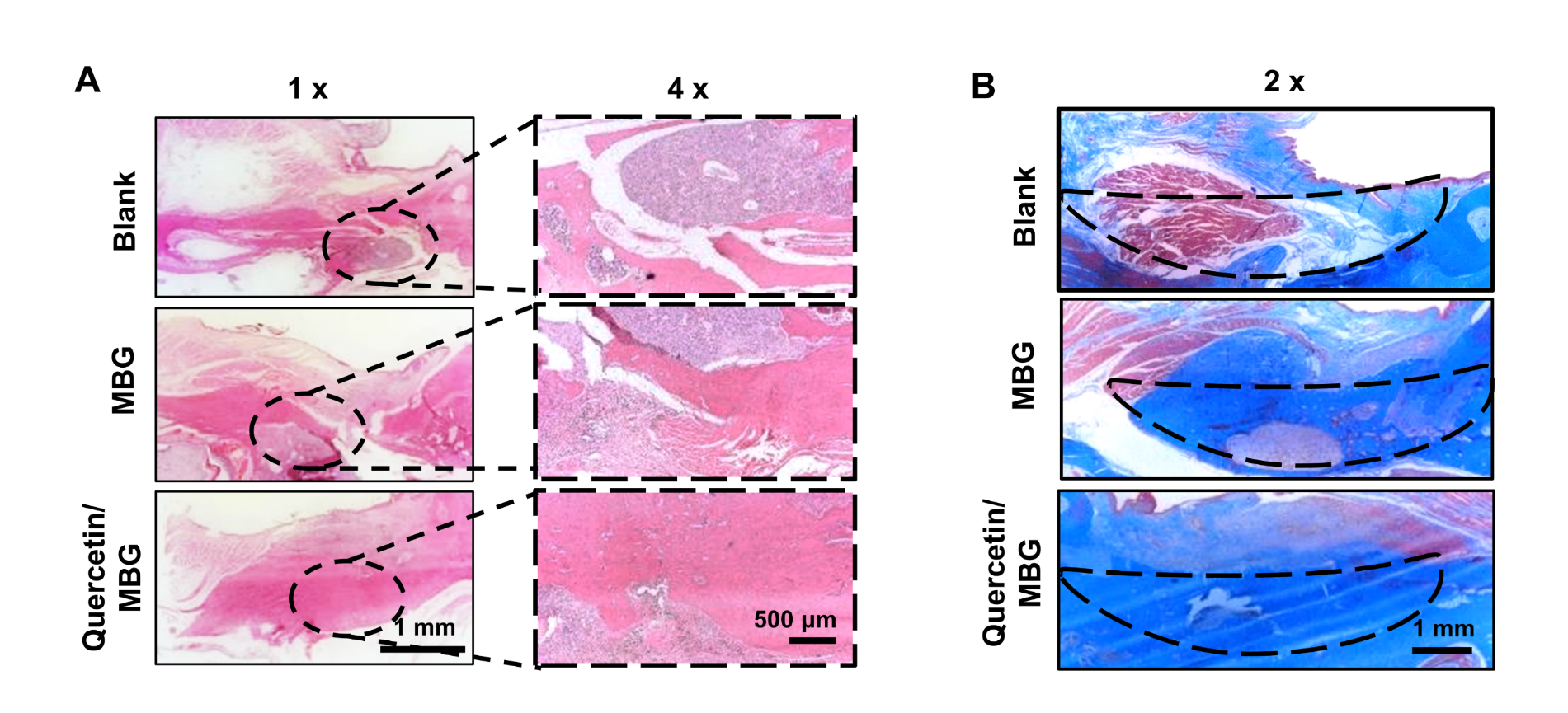
Supplementary Figures**

**Fig. S1 (A-B)** **The H＆E and Masson staining of samples collected in 8 weeks.**

**
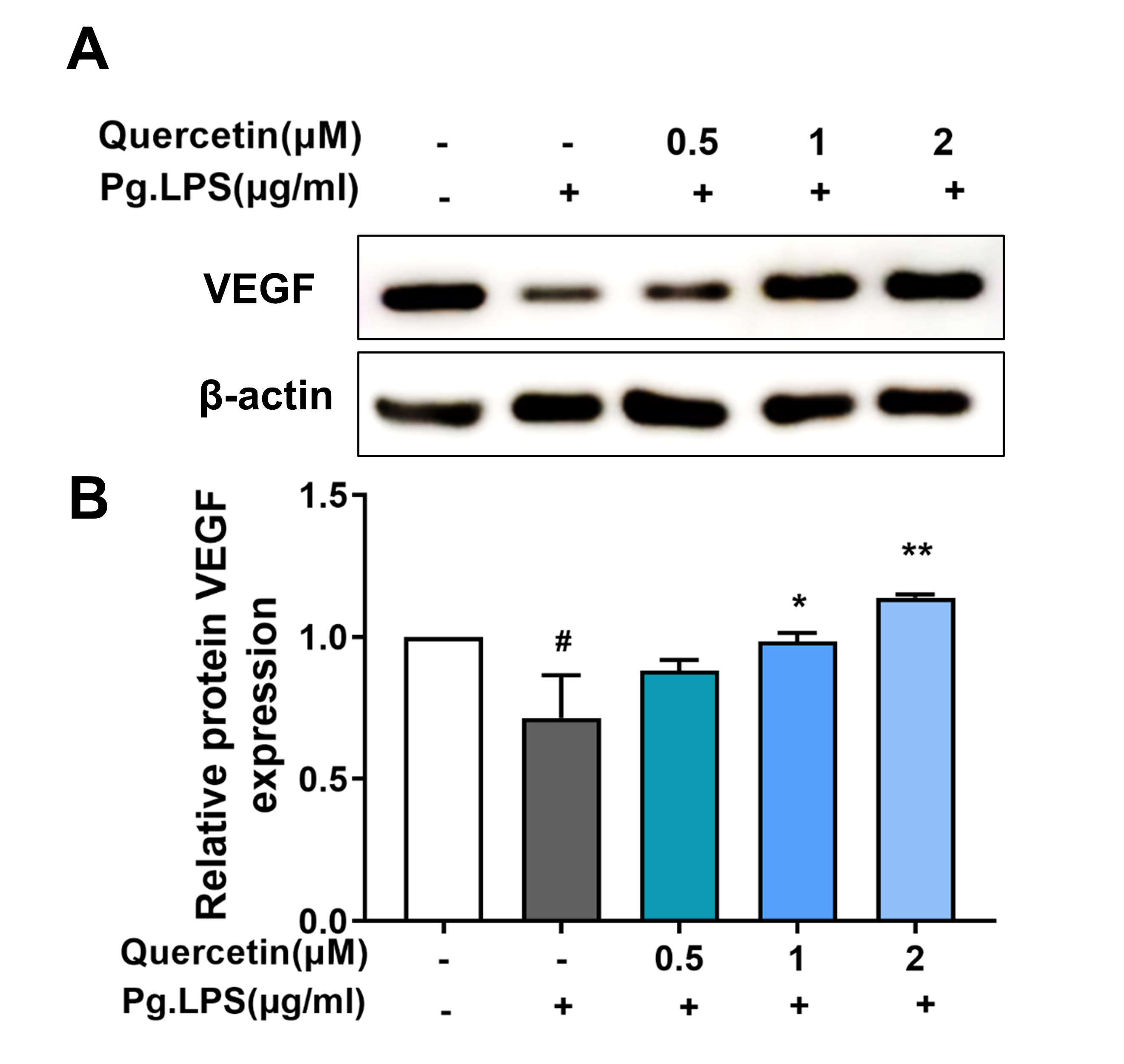
**
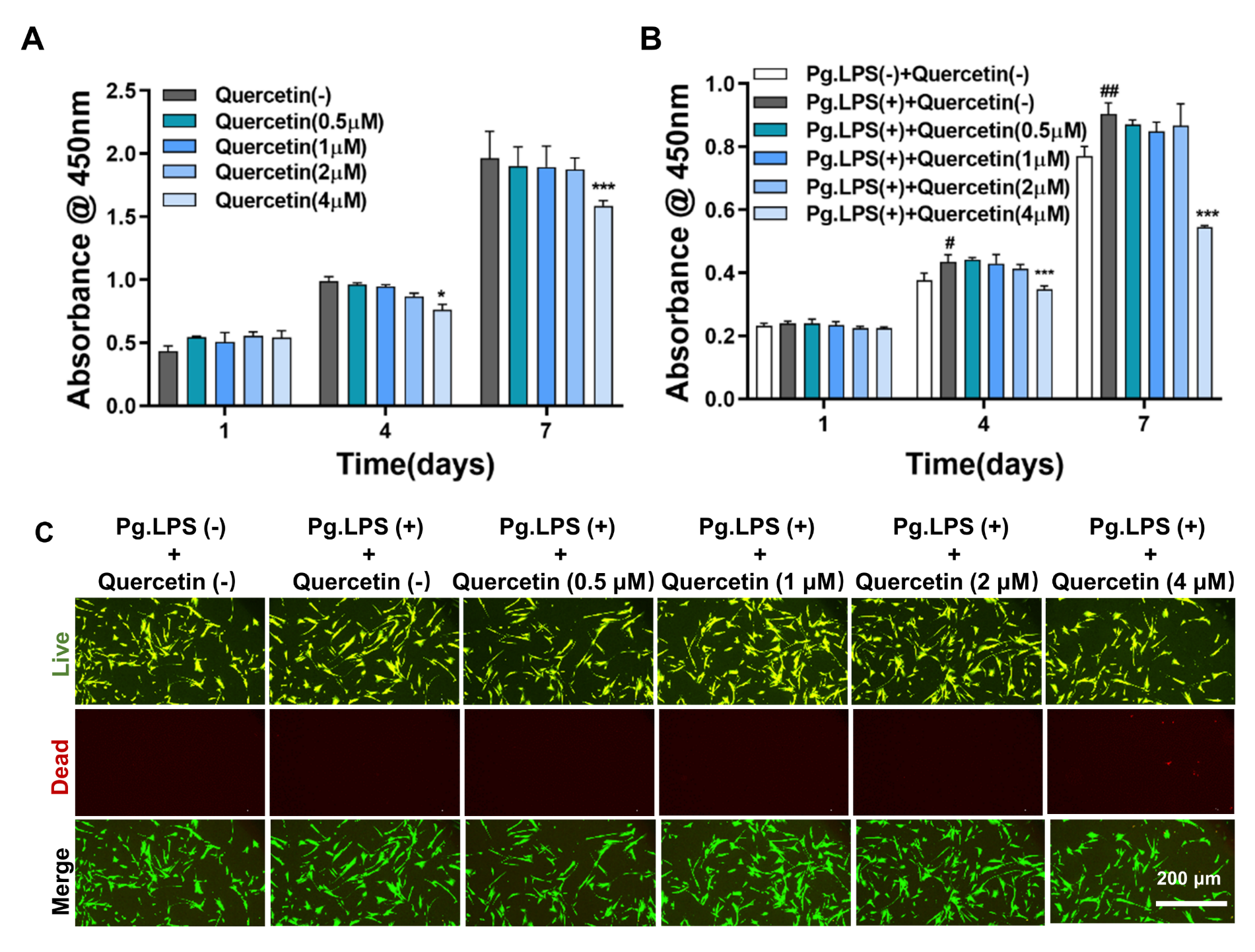
**Fig. S2 (A-B)** **Proliferation results of PDLSCs incubated with quercetin for 1,4 and 7 days in both physiological and** **periodontitis environment by using CCK-8 assay. (C) Live/Dead staining of PDLSCs on day 1 under the stimulation of quercetin under periodontitis environment. *(^#^p < 0.05, ^##^p < 0.01 and ^###^p < 0.001 compared to Pg.LPS (-) + Quercetin (-); ^*^p < 0.05, ^**^p < 0.01 and ^***^p < 0.001 compared to Pg.LPS (+) + Quercetin (-); Data re represented as the mean ± SEM, n=3)***

**Fig. S3 (A-B) The protein level of PDCD4 in PDLSCs incubated with quercetin for 7 days under periodontitis environment by western blot. *(^#^p < 0.05, ^##^p < 0.01 and ^###^p < 0.001 compared to Pg.LPS (-) +Quercetin (-); ^*^p < 0.05, ^**^p < 0.01 and ^***^p < 0.001 compared to Pg.LPS (+) +Quercetin (-); Data re represented as the mean ± SEM, n=3)***


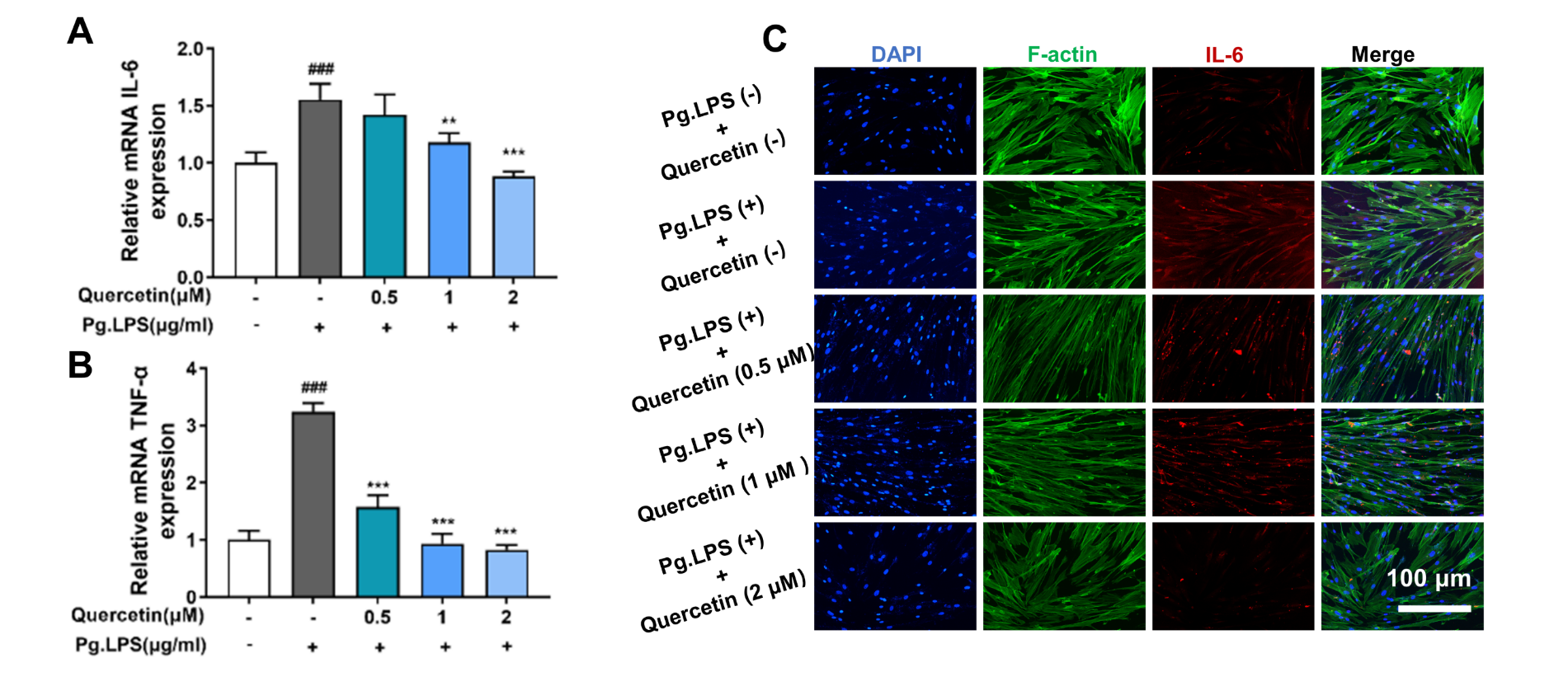


**Fig. S4 (A-C)** **The expression of inflammation-related genes and protein of PDLSCs under periodontitis microenvironment after incubation with quercetin for 1 day. *(^#^p < 0.05, ^##^p < 0.01 and ^###^p < 0.001 compared to Pg.LPS (-)* *+Quercetin (-); ^*^p < 0.05, ^**^p < 0.01 and ^***^p < 0.001 compared to Pg.LPS (+) +Quercetin (-); Data re represented as the mean ± SEM, n=3)***

**
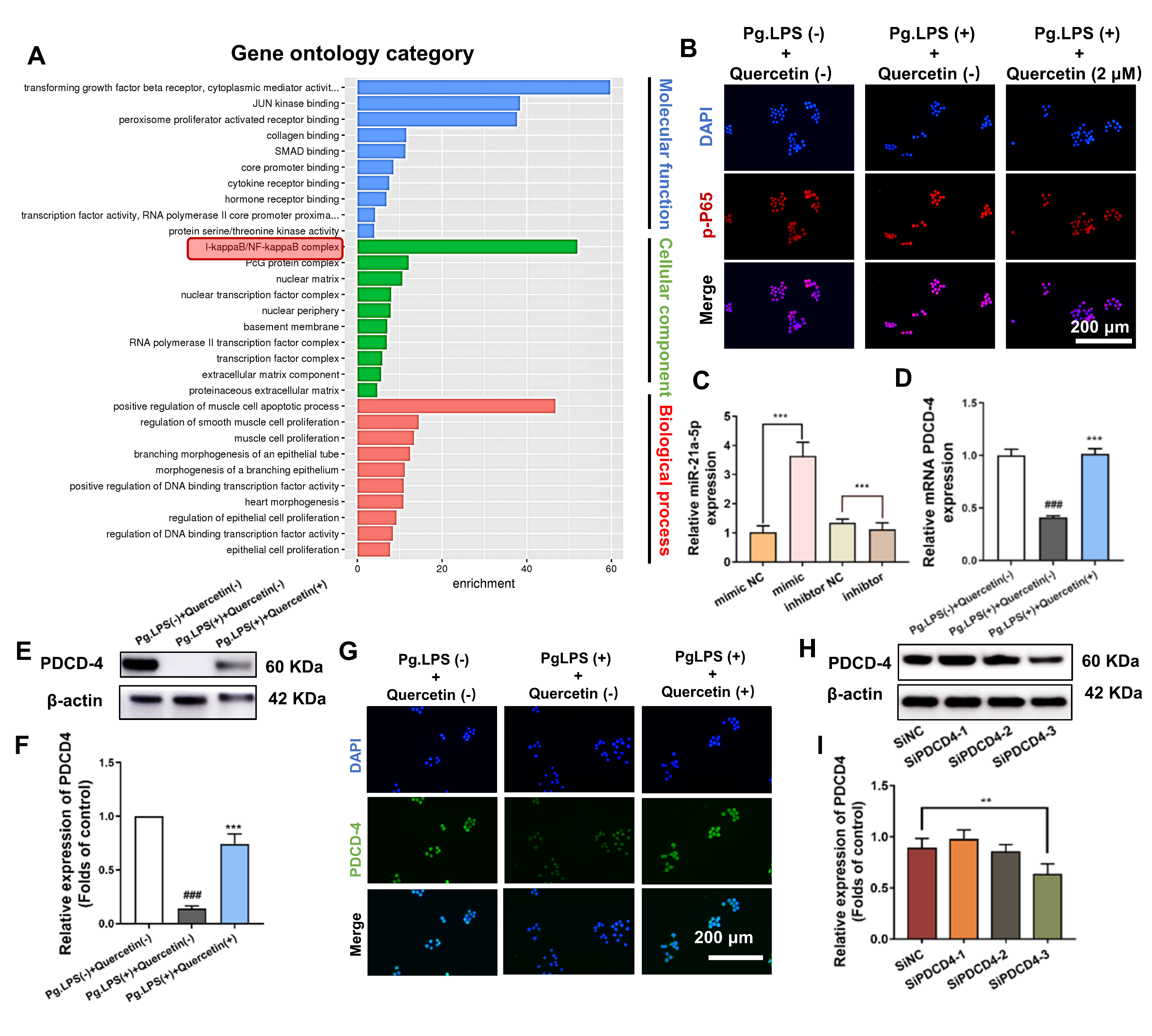
Fig. S5 (A) The Go enrichment analysis of** **Pg.LPS（+） + Quercetin（-） vs** **Pg.LPS（+） + Quercetin（+） group. (B)Immunofluorescence images of the p-P65 protein expression in macrophages. (C) The miR-21a-5p expression in RAW264.7 transfected by mir-21a-5p mimic NC, mimic, inhibitor NC and inhibitor. (D-G) qRT-PCR, western blot and immunofluorescence staining of the PDCD4 among Pg.LPS (-) +Quercetin (-), Pg.LPS (+) +Quercetin (-) and Pg.LPS (+) +Quercetin (+) groups. (H-I) The protein level of PDCD4 in siNC, siPDCD4-1-, siPDCD4-2- or siPDCD4-3-transfected macrophages determined by western blot. *(In Fig. S5 D-F, ^#^p < 0.05, ^##^p < 0.01 and ^###^p < 0.001 compared to Pg.LPS (-) + Quercetin (-) group and ^*^p < 0.05, ^**^p < 0.01 and ^***^p < 0.001 compared to Pg.LPS (+) + Quercetin (-) group. In Fig. S5 C and Fig. S6 I, ^*^p < 0.05, ^**^p < 0.01 and ^***^p < 0.001; Data are represented as the mean ± SEM, n=3)***

**
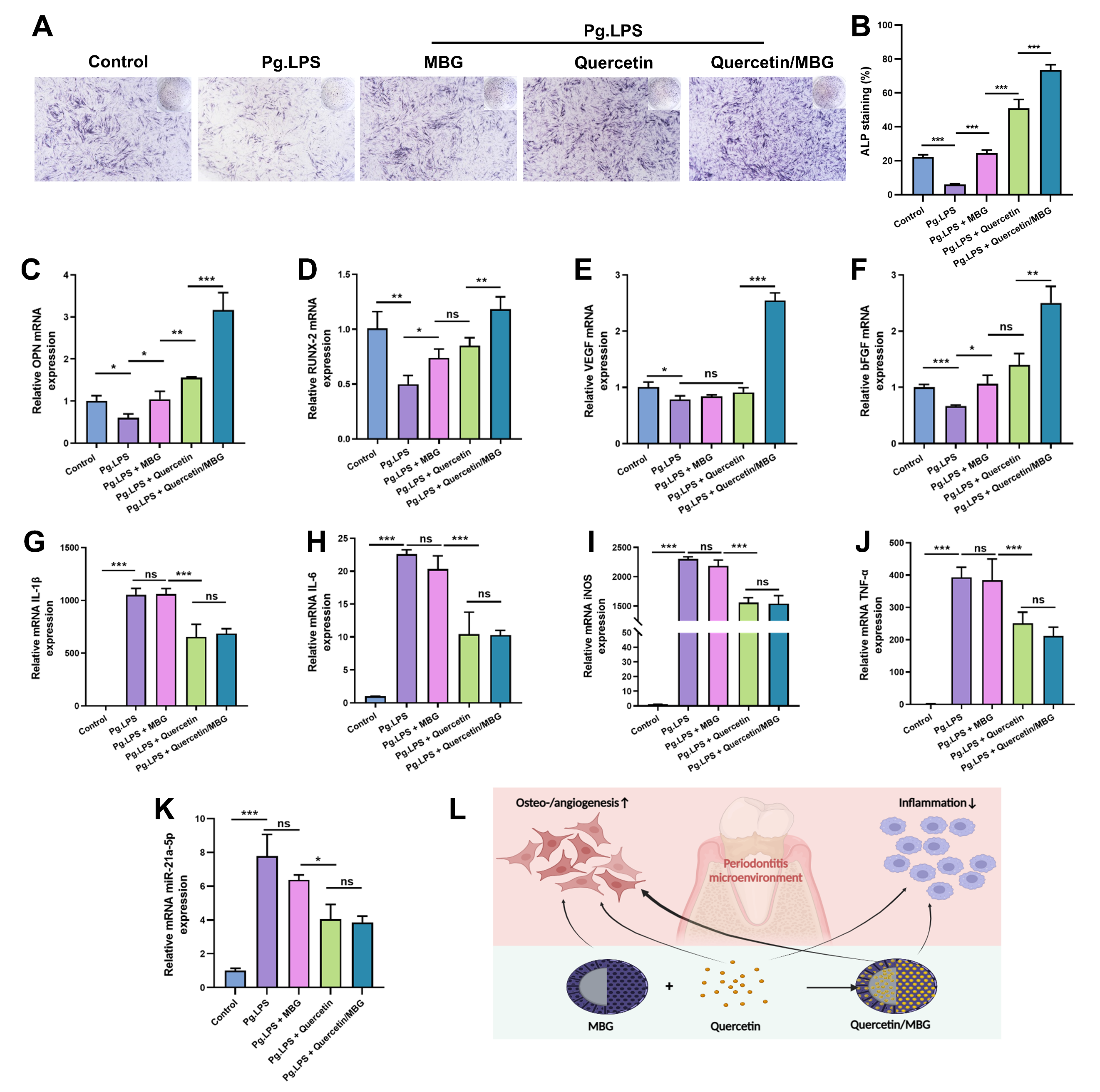
****Fig. S6 (A-B) ALP staining and its quantitative results after incubation of PDLSCs with MBG, Quercetin and Quercetin/MBG for 7 days under periodontitis microenvironment. (C-F) Effect of MBG, Quercetin and Quercetin/MBG on the expression of** **osteogenic-related genes (OPN and RUNX-2) and angiogenic-related genes (VEGF and bFGF) of PDLSCs under periodontitis microenvironment through qRT-PCR. (G-J) Effect of MBG, Quercetin and Quercetin/MBG on the expression of inflammatory genes (IL-1β，IL-6， iNOS and TNF-α) of RAW264.7 under periodontitis microenvironment through qRT-PCR. (K) Effect of MBG, Quercetin and Quercetin/MBG on the expression of miR-21a-5p** **of RAW264.7** **under periodontitis microenvironment through qRT-PCR. (L) Schematic of Quercetin/MBG on PDLSCs and RAW264.7 under periodontitis microenvironment. *(ns, no significant difference; ^*^p < 0.05, ^**^p < 0.01 and ^***^p < 0.001; Data are represented as the mean ± SEM, n=3)***

**Supplementary Reference**

1. Hu Q, Li Y, Zhao N, Ning C, Chen X. Facile synthesis of hollow mesoporous bioactive glass sub-micron spheres with a tunable cavity size. *Materials Letters*. 2014;134:130-133.

2. Casarrubios L, Gómez-Cerezo N, Feito MJ, Vallet-Regí M, Arcos D, Portolés MT. Ipriflavone-Loaded Mesoporous Nanospheres with Potential Applications for Periodontal Treatment. *Nanomaterials (Basel)*. 2020;10:2573.

3. Ren S, Zhou Y, Zheng K, Xu X, Yang J, Wang X, et al. Cerium oxide nanoparticles loaded nanofibrous membranes promote bone regeneration for periodontal tissue engineering. *Bioact Mater*. 2022;7:242-253.
